# Cytological and proteomic analyses of horsetail (*Equisetum arvense* L.) spore germination

**DOI:** 10.3389/fpls.2015.00441

**Published:** 2015-06-17

**Authors:** Qi Zhao, Jing Gao, Jinwei Suo, Sixue Chen, Tai Wang, Shaojun Dai

**Affiliations:** ^1^Development Center of Plant Germplasm Resources, College of Life and Environmental Sciences, Shanghai Normal UniversityShanghai, China; ^2^Key Laboratory of Saline-alkali Vegetation Ecology Restoration in Oil Field, Ministry of Education, Alkali Soil Natural Environmental Science Center, Northeast Forestry UniversityHarbin, China; ^3^Department of Biology, Interdisciplinary Center for Biotechnology Research, Genetics Institute, Plant Molecular and Cellular Biology Program, University of FloridaGainesville, FL, USA; ^4^Institute of Botany, Chinese Academy of SciencesBeijing, China

**Keywords:** spore germination, *Equisetum arvense* L., fern, proteomics, single cell, polar growth

## Abstract

Spermatophyte pollen tubes and root hairs have been used as single-cell-type model systems to understand the molecular processes underlying polar growth of plant cells. Horsetail (*Equisetum arvense* L.) is a perennial herb species in Equisetopsida, which creates separately growing spring and summer stems in its life cycle. The mature chlorophyllous spores produced from spring stems can germinate without dormancy. Here we report the cellular features and protein expression patterns in five stages of horsetail spore germination (mature spores, rehydrated spores, double-celled spores, germinated spores, and spores with protonemal cells). Using 2-DE combined with mass spectrometry, 80 proteins were found to be abundance changed upon spore germination. Among them, proteins involved in photosynthesis, protein turnover, and energy supply were over-represented. Thirteen proteins appeared as proteoforms on the gels, indicating the potential importance of post-translational modification. In addition, the dynamic changes of ascorbate peroxidase, peroxiredoxin, and dehydroascorbate reductase implied that reactive oxygen species homeostasis is critical in regulating cell division and tip-growth. The time course of germination and diverse expression patterns of proteins in photosynthesis, energy supply, lipid and amino acid metabolism indicated that heterotrophic and autotrophic metabolism were necessary in light-dependent germination of the spores. Twenty-six proteins were involved in protein synthesis, folding, and degradation, indicating that protein turnover is vital to spore germination and rhizoid tip-growth. Furthermore, the altered abundance of 14-3-3 protein, small G protein Ran, actin, and caffeoyl-CoA O-methyltransferase revealed that signaling transduction, vesicle trafficking, cytoskeleton dynamics, and cell wall modulation were critical to cell division and polar growth. These findings lay a foundation toward understanding the molecular mechanisms underlying fern spore asymmetric division and rhizoid polar growth.

## Introduction

Sexual reproduction is crucial in plant life cycle. Spermatophyte seeds, pollen grains, and fern spores play central roles in sexual reproduction with common capability of surviving under harsh conditions upon emergence from dormancy. They have physiological resemblance with each other as to features of resurrection and polar growth during the emergence of roots, pollen tubes, and rhizoids, respectively. Previous reports have addressed the physiological and molecular characteristics of pollen and seed germination using genomics and proteomics approaches (Dai et al., 2007a,b; Tan et al., 2013), but comparable information is lacking for fern spore germination.

The similarity of germination among spermatophyte pollen grains, seeds, and fern spores includes mobilization and organization of limited reserves for polar growth within a short time frame. Previously, 33 and 123 genes were found to be commonly expressed when *Ceratopteris richardii* spores were compared with *Arabidopsis thaliana* pollen and seeds, respectively (Bushart and Roux, 2007; Salmi et al., 2007). Some proteins encoded by these genes (e.g., *Rop GTPase, Mago nashi, calmodulin 2, No pollen germination 1, phospholipase D, synaptobrevin*, and *constitutive photomorphogenic 9*) are mainly involved in calcium signaling, vesicle trafficking, and ubiquitin-mediated protein degradation. Although the protein functions encoded by some of the important genes (e.g., *Rop GTPase, calmodulin*, and *phospholipase D*) in pollen grains have been well-studied (Malhó et al., 2006), their roles in fern spore germination are not clear. In spite of polar growth similarities, pollen and fern spores are evolutionarily distinct. The pollen grains are reduced male gametophytes with two/three cells to reach ovary by tip-growing pollen tube after recognition on appropriate stigma, while the mononuclear fern spores can generate live-independent gametophyte through germination (Dai et al., 2008). Fern spore germination is somewhat more complex in that they undergo extensive cell division and differentiation during the emergences of rhizoid and prothallus under various environmental conditions (Bushart and Roux, 2007). Fern spores represent a new single-celled model for investigating asymmetric cell division, differentiation, and polar growth (Chatterjee et al., 2000; Bushart and Roux, 2007).

Previous physiological studies have reported that spore germination of more than 200 fern species was modulated by various environmental factors, such as light, gravity, calcium, phytohormones, and temperature (Suo et al., 2015). Recently, gene function analyses revealed that several signaling pathways are crucial for fern spore germination. Phytochrome and cryptochrome signaling is important for the first spore cell mitosis (Suetsugu and Wada, 2003; Kamachi et al., 2004; Tsuboi et al., 2012). Besides, gravity and calcium signaling determines polarity establishment, cell asymmetry division, and the direction of rhizoid elongation (Chatterjee et al., 2000; Bushart and Roux, 2007; Bushart et al., 2014), and nitric oxide functions as a signal molecule in the regulation of gravity-directed fern spore polarity (Salmi et al., 2007). Moreover, gibberellin and antheridiogen can initiate and promote spore germination in many species, but abscisic acid, jasmonic acid, and ethylene have only minor promoting effects (Suo et al., 2015). In addition, the enzymes involved in glyoxylate cycle (e.g., isocitrate lyase and malate synthase) and genes encoding aconitase in the conversion from citrate to isocitrate were induced in germinating spores from *Onoclea sensibilis* (DeMaggio and Stetler, 1980), *Anemia phyllitidis* (Gemmrich, 1979), and *C. richardii* (Banks, 1999), indicating the importance of lipid reserve degradation and mobilization during spore germination. However, the molecular regulatory mechanisms in these processes are still unknown.

Germination of fern spores, especially the chlorophyllous spores (green spores), is a sophisticated signaling and metabolic process. Spores from *Equisetum* species are chlorophyll-bearing and of short viability (only a few weeks) (Ballesteros et al., 2011). *Equisetum* spores can geminate immediately under appropriate conditions with high humidity (Lebkuecher, 1997), therefore, they are good materials for studying the chlorophyllous spore germination. *Equisetum* is the oldest living genus of vascular plants, containing 15–25 extant hollow-stemmed taxa (Guillon, 2007). Most *Equisetum* species are regarded as persistent weeds in wetlands (Large et al., 2006). The reproduction of *Equisetum* species is mainly dependent on the growth of the rhizomes underground, but not the spore germination. Large-scale comparative proteomics of developing rhizomes of *Equisetum hyemale* have revealed that 1911 and 1860 proteins in rhizomes apical tip and elongation zone, respectively (Balbuena et al., 2012). However, the cellular and proteomic features of spore germination are still lacking. In the present study, we carried out cellular and 2-DE based proteomics analysis of horsetail (*Equisetum arvense* L.) spore germination to reveal the signaling and metabolic characteristics.

## Materials and methods

### Collection and germination of mature horsetail spores

The mature fertile sporophylls (the separate stalks in the spring) of horsetail (*E. arvense*) were collected in suburb of Harbin (45° 27′ N, 127° 52′ E), Heilongjiang province, China. The mature spores (MS) were released by its elaters moving from the sporangia during dehydration at room temperature (25°C). To synchronize spore germination, the collected fresh MS were soaked in deionized H_2_O overnight in the dark. Then, the rehydrated spores (RS) were transferred into a liquid germination medium (4.6 mM Ca(NO_3_)_2_·4H_2_O, 2 mM KNO_3_, 1.5 mM KH_2_PO_4_, 0.8 mM MgSO_4_·7H_2_O) and cultured in an environmentally controlled chamber at 25 ± 2°C, 24 h light at 60 μmol·m^−2^ · s^−1^. The amount of germinating spores at various stages of RS, double-celled spores (DCS), germinated spores (GS), and spores with protonemal cells (SPC) was examined under a microscope. All these spores were collected by centrifugation at 500 × *g* for 5 min, and used immediately after collection for protein extraction or storage at −80°C.

### Observation of spore morphology upon germination

Morphological characteristics of spores upon germination were examined under a Axioskop 40 fluorescence microscope (Zeiss, Oberkochen, Germany) without or with staining using 0.2 μg·μL^−1^ 4,6-diamidino-2-phenylindole (Molecular Probes, Carlsbad, USA). Observation of MS was performed under HITACHI S-520 scanning electron microscope (Hitachi, Tokyo, Japan) (Dai et al., 2002). Living MS were prepared by standard techniques for scanning electron microscope observation. Spores were fixed, gradually dehydrated, and critically point dried by Balt-Tec CPD-030 critical point dryer (Balt-Tec AG, Balzers, Liechtenstein) according to Dai et al. (2002). Dry spores were mounted on aluminum stubs using double-sided tape and coated with gold-palladium using a Denton Vacuum Desk II sputter coater (Denton Vacuum Inc., Cherry Hill, USA).

### Protein extraction and quantification

For protein preparation, 0.5 g spores in the five stages of germination (MS, RS, DCS, GS, and SPC) were ground to powder in liquid nitrogen using chilled mortar and pestle. Total protein of spores was extracted according to the method of Wang et al. (2010). Protein samples were prepared independently from three different batches of plants, considered as three biological replicates. Protein concentration was determined using a Quant-kit according to manufacture's instructions (GE Healthcare, Salt Lake City, USA).

### 2-DE, gel staining and protein abundance analysis

The protein samples were separated and visualized using 2-DE according to Dai et al. (2006). An aliquot of 1.3 mg total protein was diluted with rehydration buffer (7 M urea, 2 M thiourea, 0.5% CHAPS, 20 mM DTT, 0.5% immobilized pH gradient IPG buffer 4–7, and 0.002% bromphenol blue) to a final volume of 450 μL and loaded onto an IPG strip holder containing a 24 cm, pH 4–7 linear gradient IPG strip (GE Healthcare, Salt Lake City, USA). Isoelectric focusing was performed in the Ettan IPGphor isoelectric focusing system (GE Healthcare, Salt Lake City, USA) following the protocol of the manufacturer. For SDS-PAGE, the equilibrated IPG strips were transferred onto 12.5% acrylamide gels by using an Ettan DALT Six Electrophoresis Unit (GE Healthcare, Salt Lake City, USA). The gels were stained by Coomassie Brilliant Blue. Gel image acquisition and analysis were conducted as previously described (Wang et al., 2010). Images were acquired by scanning each stained gel using an ImageScanner (GE Healthcare, Salt Lake City, USA) at a resolution of 300 dpi and 16-bit grayscale pixel depth, and then analyzed using ImageMaster 2D version 5.0 (GE Healthcare, Salt Lake City, USA). The experimental molecular weight of each protein was estimated by comparison with the coseparated molecular weight markers. The experimental pI of each protein was determined by its migration on IPG linear strips. For quantitative analysis, the average vol% values were calculated from three biological replicates. Protein spots with reproducible and statistically significant changes in intensity (greater than 1.5-fold and *p* < 0.05) were considered to be differentially abundant protein (DAP) spots.

### Protein identification using mass spectrometry and database searching

The DAP spots were manually excised from the 2-DE gels, and the in-gel digestion was performed as described previously (Dai et al., 2006). Tandem mass spectrometry spectra were acquired on a ESI-Q-TOF mass spectrometry (QSTAR XL) and a ESI-Q-Trap mass spectrometry (Applied Biosystems, Foster City, USA) (Wang et al., 2010; Yu et al., 2011). The tandem mass spectrometry spectra were searched against the NCBI non-redundant protein database (http://www.ncbi.nlm.nih.gov/) using Mascot software (Matrix Science, London, UK), according to the searching criteria described previously (Yu et al., 2011). The taxonomic category was green plants (3,019,757 sequence entries), mass accuracy was 0.3 Da, and the maximum number of missed cleavages was set to one. To obtain highly confident identification, proteins had to meet the following criteria: (1) the top hits on the database searching report, (2) a probability-based MOWSE score greater than 43 (*p* < 0.05), and (3) more than two peptides matched with nearly complete y-ion series and complementary b-ion series. The mass spectrometry proteomics data have been deposited to the ProteomeXchange Consortium (Vizcaíno et al., 2014) via the PRIDE partner repository with the dataset identifier PXD002218.

### Protein classification and hierarchical clustering analysis

For function classification of DAPs, the peptide sequences of each DAP were aligned against the Gene Ontology (GO) protein database (http://geneontology.org) following the policies and procedures provided by the GO Consortium (http://geneontology.org/). The proteins were classified according to their cellular component, molecular function, and biological process. Also, PSI and PHI-BLAST programs (http://www.ncbi.nlm.nih.gov/BLAST/) were used to search against the NCBI non-redundant protein database for protein functional domain annotation. Besides, the biological function of protein was obtained from the Kyoto Encyclopedia of Genes and Genomes pathway database (http://www.kegg.jp/kegg/). In addition, the conservative protein function during spore germination was predicted from previous publications on the germinating seeds and pollen. Finally, by integrative analysis of all the information collected from aforementioned processes, each DAP was classified into certain functional category defined by us. The definition of functional category is referred from literatures on pollen and seed germination (Supplementary Figure S1). Log (base 2) transformed ratios were used for hierarchical clustering analysis using Cluster 3.0 available on the Internet (http://bonsai.hgc.jp/~mdehoon/software/cluster/software.htm). Protein abundance ratio was calculated as protein abundance at MS stage divided by abundance at each stage. Using a tree algorithm, these DAPs were organized based on similarities in the expression profile. These proteins can be joined by very short branches if they are very similar to each other, and by increasingly longer branches as their similarity decreases. Java TreeView (http://jtreeview.sourceforge.net/) was used for data visualization.

### Protein subcellular location and protein-protein interaction analysis

The subcellular location of the identified proteins was predicted using five internet tools: (1) YLoc (http://abi.inf.uni-tuebingen.de/Services/YLoc/webloc.cgi), confidence score ≥0.4; (2) LocTree3 (https://rostlab.org/services/loctree3/), expected accuracy ≥80%; (3) ngLOC (http://genome.unmc.edu/ngLOC/index.html), probability ≥80%; (4) TargetP (http://www.cbs.dtu.dk/services/TargetP/), reliability class ≤3; (5) Plant-mPLoc (http://www.csbio.sjtu.edu.cn/bioinf/plant-multi/), no threshold value in Plant-mPLoc. Only the consistent predictions from at least two tools were accepted as a confident result. For the inconsistent prediction results among five tools, subcellular localizations for corresponding proteins were predicted based on literatures.

The protein-protein interactions were predicted using the web-tool STRING 9.1 (http://string-db.org). The DAPs homologs in Arabidopsis were found by sequence BLASTing in TAIR database (http://www.arabidopsis.org/Blast/index.jsp). The homologs were subjected to the molecular interaction tool of STRING 9.1 for creating the proteome-scale interaction network.

### Statistical analysis

All the results were presented as means ± standard deviation of three biological replicates. Data were analyzed by One-Way ANOVA using the statistical software SPSS 17.0 (SPSS Inc., Chicago, USA). The mean values from different stages of spore germination were compared by least significant difference *post-hoc* test. A *p*-value less than 0.05 was considered statistically significant.

## Results and discussion

### Horsetail chlorophyllous spore germination process

**Figure 1:**
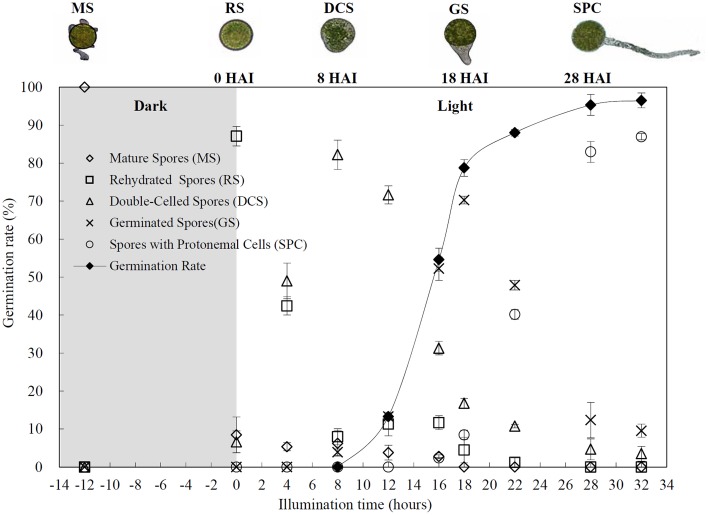
**Germination time course of spores from** ***E. arvense***. Mature spores (MS) (open rhombuses) were cultured on Knop's medium. After 12 h dark imbibition, 87% of the rehydrated spores (RS) (open squares) completed rehydration, which were enlarged with an open trilete aperture. Then, spores were cultured under continuous illumination. At 8 h after illumination (HAI), 82% spores completed first mitosis to form double-celled spores (DCS) (open triangles). At 18 HAI, over 70% spores generated rhizoids, which were defined as germinated spores (GS) (crosses). At 32 HAI, more than 87% spores finished the second cell division, which were called spores with protonemal cells (SPC) (open circles). The spore germination rate (filled rhombuses) was increased gradually in this time course, and the maximum germination rate was over 95% at the stage of SPC. Error bar indicates ± standard deviation.

Chlorophyllous spores from fern species in a few unrelated taxa in Pteridophyta can germinate in less than 3 days and their viability lasts about 1 year or less (Lloyd and Klekowski, 1970). *Equisetum* spores are typical chlorophyllous spores with short viability, which can germinate immediately under humid conditions and remain viable for about 2 weeks (Lebkuecher, 1997). In this study, the spores were sown and cultured on Knop's medium under two different illumination levels (i.e., 30 μmol·m^−2^ · s^−1^ and 60 μmol·m^−2^ · s^−1^) after 12 h dark imbibition in deionized H_2_O at room temperature. We found that the light illumination level has obvious effects on horsetail spore germination rate. At 16 h after illumination (HAI), the germination rate of spores under higher illumination level (60 μmol·m^−2^ · s^−1^) was over 50%, but the germination rate of spores under lower illumination level (30 μmol·m^−2^ · s^−1^) was only 20%. The spores under the higher light intensity reached to the maximum germination rate of 95% at 32 HAI, but the maximum germination rate of spores under 30 μmol·m^−2^ · s^−1^ was only 35% (Supplementary Figure S2). Thus, the 60 μmol·m^−2^ · s^−1^ light was used for horsetail spore germination. Actually, the horsetail MS have initiated germination during the process of dark imbibition. Eighty-seven percent of the RS finished their cell nucleus polar migration toward the gravity, and the nucleus of spore started their division at 12 HAI for the preparation of asymmetry cell mitosis (Figure 1). At 8 HAI, 82% spores finished the first mitosis to generate DCS containing a larger cell and a smaller cell, and the smaller cell of 4% spores elongated to form an obvious rhizoid. At this time point, we started to calculate the spore germination rate that is defined as the ratio of GS number to total spore number. At 18 HAI, more than 70% of spores generated the rhizoids, being defined as GS. At 32 HAI, spores obtained the maximum germination rate of over 95%, and the larger cell (protonemal cell) of over 87% spores have finished the second mitosis to give rise to the photosynthetic prothallus. The spores at this stage were defined as SPC (Figure 1).

### Cytological characteristics of horsetail germinating spores

**Figure 2:**
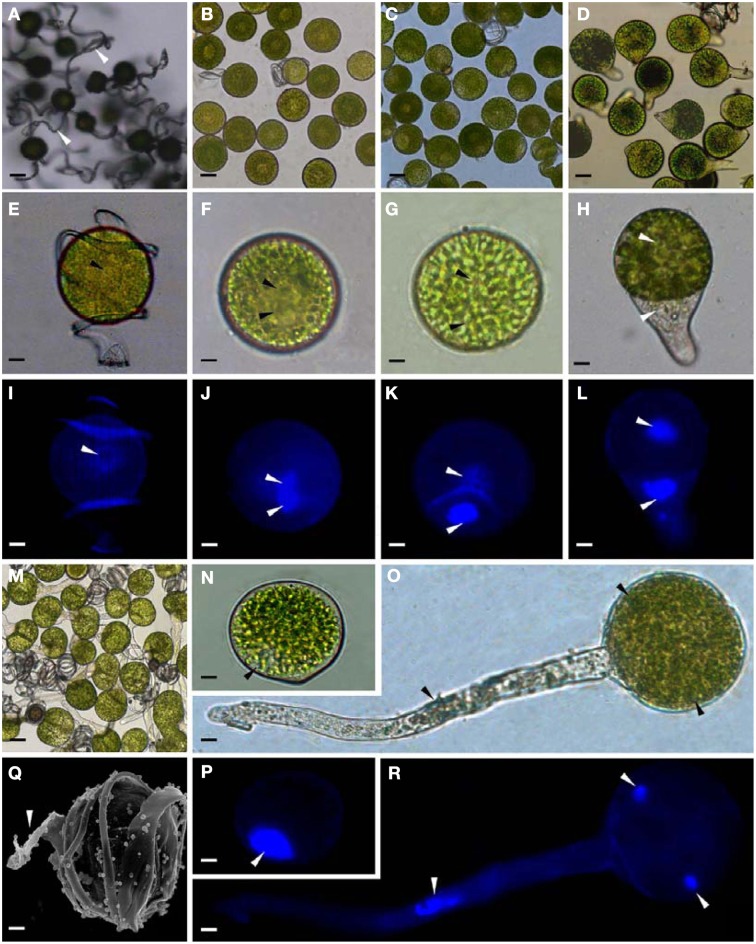
**Morphology of mature and germinating spores from** ***E. arvense***. **(A,E,I,N,P,Q)** Mature spores (MS). **(A)** MS groups, arrows show elaters, bar = 20 μm; **(E,I)** nucleus center-localized MS with elaters under bright-field microscope (BFM) and fluorescence microscope (FM), arrows show nuclei, bar = 5 μm; **(N,P)** nucleus-migrated MS without elaters under BFM and FM, arrows show nuclei, bar = 5 μm; **(Q)** MS with elaters under scanning electron microscope, arrows show elaters, bar = 8 μm. **(B,F,J)** Rehydrated spores (RS). **(B)** RS groups, bar = 15 μm; **(F,J)** RS under BFM and FM, arrows show nuclei, bar = 5 μm. **(C,G,K)** Double-celled spores (DCS). **(C)** DCS groups, bar = 15 μm; **(G,K)** DCS under BFM and FM, arrows show nuclei, bar = 5 μm. **(D,H,L)** Germinated spores (GS). **(D)** GS groups, bar = 15 μm; **(H,L)** GS under BFM and FM, arrows show nuclei, bar = 5 μm. **(M,O,R)** Spores with protonemal cells (SPC). **(M)** SPC groups, bar = 15 μm; **(O,R)** SPC under BFM and FM, arrows show nuclei, bar = 5 μm.

The horsetail chlorophyllous spores were generated in sporangium from strobili. The MS were released from partially dried strobilus. Horsetail spores are unusual in the morphological and physiological aspects. The spores are typically about 25–35 μm in diameter with four flexible ribbon-like elaters about 100 μm each (Figures 2A,E,I,Q). The elaters initially wrapping around the spore body can deploy upon dehydration and fold back in humid air. The elater movement driven by humidity variations led to the spore exiting from sporangium, and especially can catch the wind again when they were jumping from the ground, which is believed to be a novel type of efficient self-propelled dispersal mechanism (Marmottant et al., 2013). The single nuclear was visible clearly in the center of the spores (Figures 2E,I). The elaters were lost when spores were cultured in liquid medium and cell nucleus has migrated toward gravity (Figures 2N,P). After 12 h dark imbibition, the RS were swelled to the diameter of about 50 μm. In RS, the nucleus has completed the first mitosis and two nuclei in the bottom side of the spore can be clearly observed (Figures 2B,F,J). Subsequently, at 8 HAI, the novel cell wall was formed between the two nuclei to generate an asymmetry DCS, containing a larger cell and a smaller cell (Figures 2C,G,K). The smaller cell started to elongate out of the spore and an approximate 30 μm long rhizoid emerged at 18 HAI to form GS (Figures 2D,H,L). In the GS, the cell nucleus of rhizoid still left inside the spores (Figures 2H,L). As rhizoid elongated, its nucleus was moving outside from the spore to the center of the rhizoid at 28 HAI (Figures 2O,R). At the same time, the larger cell inside the spore can complete the second mitosis to form a new protonemal cell (Figures 2O,R). Thus, the spore finish its germination by the formation of spores with a rhizoid cell and two protonemal original cells (Figures 2M,O,R).

### DAPs upon spore germination

**Figure 3:**
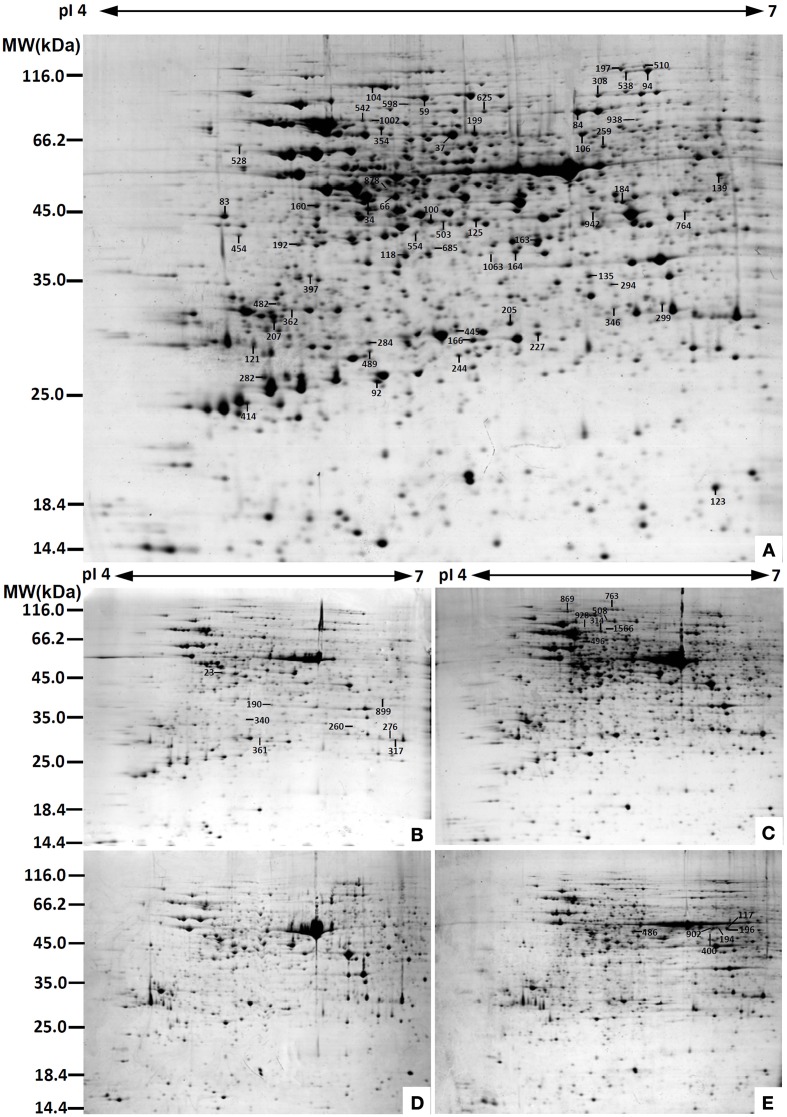
**Representative 2-DE images of proteins in various germination stages from** ***E. arvense*** **spores. (A)** Mature spores. **(B)** Rehydrated spores. **(C)** Double-celled spores. **(D)** Germinated spores. **(E)** Spores with protonemal cells. Proteins were separated on 24 cm IPG strips (pH 4–7 linear gradient) using IEF in the first dimension, followed by 12.5% SDS-PAGE gels in the second dimension. The 2-DE gel was stained with Coomassie Brilliant Blue. A total of 80 differentially abundant proteins identified by ESI-Q-TOF and ESI-Q-Trap tandem mass spectrometry are marked with numbers on the gels. Molecular weight (MW) in kDa and pI of proteins are indicated on the left and top of the gels, respectively. Detailed information can be found in Table 1 and Supplementary Table S1.

To determine DAPs in mature and germinating spores, protein samples collected at the five stages (MS, RS, DCS, GS and SPC) were subjected to 2-DE analysis. On the Coomassie Brilliant Blue-stained gels (pH 4–7, 24 cm IPG srtip), 1243 ± 47, 1247 ± 34, 1254 ± 31, 1229 ± 57, and 1234 ± 40 spots from MS, RS, DCS, GS, and SPC were detected, respectively (Figure 3, Supplementary Figure S3). Among them, 139 protein spots showed differential abundances in five distinct stages of spore germination (>1.5-fold, *p* < 0.05). A total of 131 spots were identified using tandem mass spectrometry and Mascot database searching, and eight spots were not matched in database. Among the proteins, 28 spots contained only single peptide match, which were considered as un-identified according to our criteria. In the remaining 103 spots, 80 spots contained a single protein each (Table 1, Supplementary Table S1) and 23 spots contained more than one protein each (Supplementary Table S2). Thus, the 80 proteins were DAPs during spore germination.

**Table 1:** **Differentially abundant proteins during** ***E. arvense*** **spore germination**.

**Spot No.^(^^a^^)^**	**Protein name^(^^b^^)^**	**Subcellular location^(^^c^^)^**	**Plant species^(^^d^^)^**	**Gi No.^(^^e^^)^**	**Thr. MW(Da)/pI^(^^f^^)^**	**Exp. MW(Da)/pI^(^^g^^)^**	**Cov (%)^(^^h^^)^**	**Sco^(^^i^^)^**	**QM^(^^j^^)^**	**V% ± SD^(^^k^^)^ MS RS DCS GS SPC**
**PHOTOSYNTHESIS (17)**										
121	Chlorophyll a/b binding protein (CAB)	Chl	*Hedera helix*	12,582	20,759/4.83	25,376/4.71	10	57	3	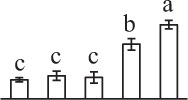
346	Ribulose-1,5-bisphosphate carboxylase/oxygenase large subunit (RBCL)	Chl	*Eryngium bourgatii*	1,292,976	53,093/5.56	28,686/6.28	6	59	3	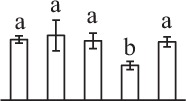
763	Ribulose-1,5-bisphosphate carboxylase/oxygenase large subunit (RBCL)	Chl	*Donatia fascicularis*	1,304,292	49,896/6.32	134,736/5.48	8	55	5	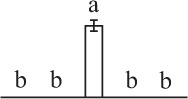
902	Ribulose-1,5-bisphosphate carboxylase/oxygenase large subunit (RBCL)	Chl	*Grammitis diminuta*	340,031,166	45,938/6.26	50,502/6.31	5	61	2	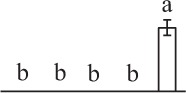
117	Ribulose-1,5-bisphosphate carboxylase/oxygenase large subunit (RBCL)	Chl	*Equisetum telmateia*	16,565,336	48,750/6.26	52,974/6.47	15	57	7	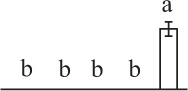
196	Ribulose-1,5-bisphosphate carboxylase/oxygenase large subunit (RBCL)	Chl	*E. telmateia*	16,565,336	48,750/6.26	51,502/6.47	16	52	6	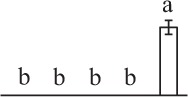
194	Ribulose-1,5-bisphosphate carboxylase/oxygenase large subunit (RBCL)	Chl	*E. telmateia*	16,565,336	48,750/6.26	51,418/6.33	13	53	6	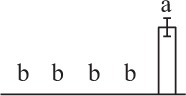
400	Ribulose-1,5-bisphosphate carboxylase/oxygenase large subunit (RBCL)	Chl	*E. telmateia*	16,565,336	48,750/6.26	49,452/6.31	11	53	5	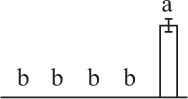
23	Ribulose-1,5-bisphosphate carboxylase/oxygenase large subunit (RBCL)	Chl	*E. bourgatii*	1,292,976	53,093/5.56	50,872/5.02	4	78	2	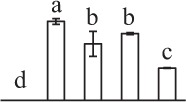
528	Ribulose-1,5-bisphosphate carboxylase/oxygenase large subunit (RBCL)	Chl	*Equisetum arvense*	1,352,773	52,493/5.86	67,620/4.64	7	60	3	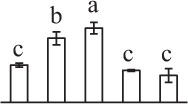
100	Ribulose-1,5-bisphosphate carboxylase/oxygenase large subunit (RBCL)	Chl	*Isoetes capensis*	83,032,384	47,561/6.30	42,425/5.47	6	55	3	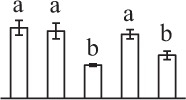
496	Ribulose-1,5-bisphosphate carboxylase/oxygenase large subunit-binding protein subunit beta (RBP)	Chl	*Solanum lycopersicum*	460,379,814	64,527/5.46	71,249/5.21	4	61	2	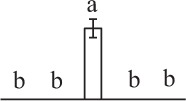
34	Ribulose-1,5-bisphosphate carboxylase/oxygenase activase (RCA)	Chl	*Gossypium hirsutum*	12,620,883	48,609/5.06	48,999/5.19	11	98	4	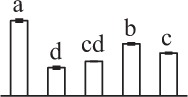
184	Ribulose-1,5-bisphosphate carboxylase/oxygenase activase (RCA)	Chl	*Hordeum vulgare*	100,614	47,496/5.64	47,356/6.31	11	94	4	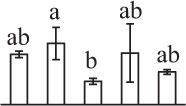
125	Ribulose-1,5-bisphosphate carboxylase/oxygenase activase (RCA)	Chl	*Musa acuminata* subsp. *malaccensis*	695,062,479	47,898/6.18	42,859/5.67	8	75	3	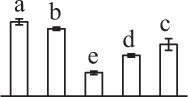
308	Transketolase (TK)	Chl	*Spinacia oleracea*	2,529,342	80,744/6.20	95,817/6.20	8	156	5	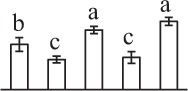
84	Transketolase (TK)	Chl	*S. lycopersicum*	460,388,792	80,615/6.26	85,056/6.11	8	90	7	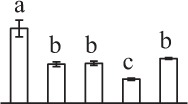
**CARBOHYDRATE AND ENERGY METABOLISM (9)**										
685	Unknown, pyruvate dehydrogenase E1 component subunit beta^*^ (PDH)	Chl	*Glycine max*	255,647,166	44,596/6.28	27,410/6.46	4	60	2	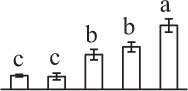
160	Malate dehydrogenase (MDH)	^#^Chl, Cyt, Mit, Pox	*Arabidopsis thaliana*	15,219,721	35,890/6.11	46,411/4.98	12	74	3	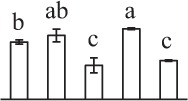
503	Malate dehydrogenase (MDH)	^#^Chl, Cyt, Mit, Pox	*A. thaliana*	15,219,721	35,890/6.11	42,425/5.53	6	57	2	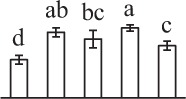
260	Malate dehydrogenase (MDH)	^#^Chl, Cyt, Mit, Pox	*A. thaliana*	11,133,509	35,548/6.11	29,729/6.41	6	59	3	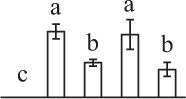
164	Malate dehydrogenase (MDH)	^#^Chl, Cyt, Mit, Pox	*Beta vulgaris* subsp. *vulgaris*	731,361,010	41,677/5.74	36,353/5.84	12	193	4	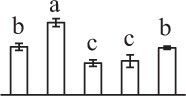
163	Malate dehydrogenase (MDH)	Chl	*Brachypodium distachyon*	357,147,942	41,864/6.97	38,560/5.94	10	60	4	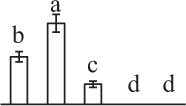
486	Enolase	Cyt	*Tarenaya hassleriana*	729,317,446	51,639/5.91	48,823/5.62	3	54	2	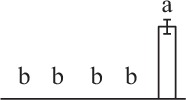
489	6-phosphogluconate dehydrogenase (6-PGDH)	^#^Chl, Cyt	*G. max*	356,513,305	54,116/6.25	24,817/5.21	12	53	5	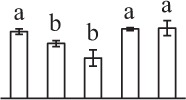
192	Fructokinase (FK)	Chl	*Lycopersicon esculentum*	23,476,263	40,620/5.41	37,710/4.91	8	129	3	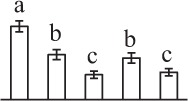
**OTHER METABOLISMS (5)**										
135	Enoyl-acyl carrier protein reductase (EAR)	Chl	*Oryza sativa* subsp. *japonica*	75,225,229	39,277/8.81	32,651/6.16	5	21	2	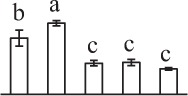
197	Hypothetical protein PHAVU_001G035500g, glycine decarboxylase^*^ (GDC)	Mit	*Phaseolus vulgaris*	593,795,946	116,167/6.65	117,088/6.30	11	157	12	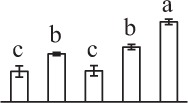
362	3-isopropylmalate dehydrogenase (IPMDH)	Chl	*A. thaliana*	15,241,338	44,305/5.75	28,512/4.87	23	67	7	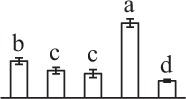
445	Hypothetical protein SELMODRAFT_406755, containing cd00517 ATP-sulfurylase domain^*^ (ATPS)	Chl	*Selaginella moellendorffii*	302,763,978	56,179/6.69	26,370/5.59	5	60	3	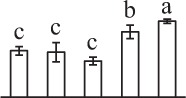
190	Predicted protein, pyridoxal biosynthesis protein PDX1^*^	Cyt	*Physcomitrella patens*	168,019,502	33,769/6.03	29,893/5.82	15	324	8	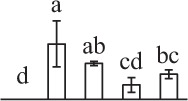
**SIGNALING AND VESICLE TRAFFICKING (8)**										
282	Unknown, containing PLN02804 chalcone isomerase domain^*^ (CHI)	Chl	*Picea sitchensis*	116,784,316	23,688/5.23	23,141/4.75	7	56	2	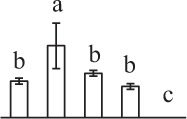
1063	Hypothetical protein SELMODRAFT_151778, containing cd00200 WD40 domain^*^ (WD40)	Nuc	*S. moellendorffii*	302,791,020	37,267/5.65	36,189/5.74	6	84	2	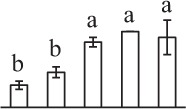
207	14-3-3 protein	Cyt	*S. oleracea*	440,573,600	30,029/4.84	27,277/4.79	16	79	6	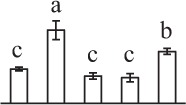
276	Unknown, containing cd00877 Ran GTPase domain^*^ (RAN)	Nuc	*P. sitchensis*	116,794,384	25,374/6.30	27,727/6.65	20	52	4	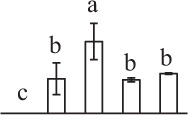
317	GTP-binding nuclear protein Ran/TC4 (RAN)	Nuc	*Vicia faba*	585,783	25,274/6.39	27,750/6.73	27	187	6	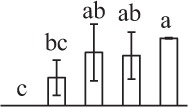
340	Unknown, containing cd00877 Ran GTPase domain^*^ (RAN)	Nuc	*P. sitchensis*	116,794,384	25,374/6.30	30,115/5.59	24	53	5	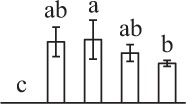
764	Unknown, containing cd00877 Ran GTPase domain^*^ (RAN)	Nuc	*P. sitchensis*	116,794,384	25,374/6.30	44,900/6.58	30	51	6	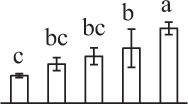
123	ADP-ribosylation factor (ARF)	Gol	*Chlamydomonas reinhardtii*	1,703,374	20,747/6.92	19,121/6.71	29	92	4	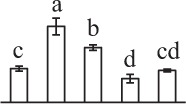
**CELL STRUCTURE (5)**										
397	Os03g0718100, actin^*^	Cyt	*O. sativa* subsp. *japonica*	115,454,971	42,014/5.30	32,585/4.95	9	82	3	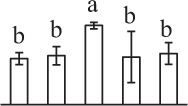
554	Reversibly glycosylated polypeptide (RGP)	Cyt	*Ricinus communis*	223,546,230	41,557/5.82	39,973/5.41	14	105	5	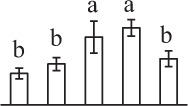
118	Reversibly glycosylated polypeptide (RGP)	Cyt	*Solanum tuberosum*	34,582,499	42,146/5.71	35,827/5.36	24	74	8	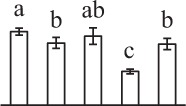
299	Unknown protein, rhamnose biosynthetic enzyme 1^*^ (RBE)	^#^Cyt	*A. thaliana*	8,493,590	33,861/5.73	29,119/6.48	7	127	4	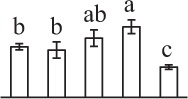
482	Caffeoyl-CoA O-methyltransferase (CCoAOMT)	^#^Cyt	*Eucalyptus gunnii*	3,023,419	28,010/5.02	29,0934.81	11	73	2	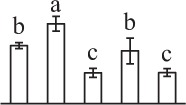
**CELL CYCLE (2)**										
104	Cell division cycle protein 48 homolog (CDC48)	Cyt	*T. hassleriana*	729,396,339	89,888/5.09	103,252/5.22	21	119	14	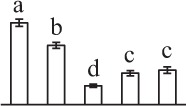
139	Proliferation-associated protein 2G4-like (PA2G4)	Nuc	*Nicotiana tomentosiformis*	697,180,533	43,837/5.96	55,758/6.73	5	52	2	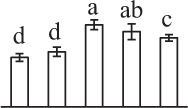
**TRANSCRIPTION RELATED (1)**										
938	Predicted protein, containing cd00771 threonyl-tRNA synthetase class II core catalytic domain^*^ (ThrRS)	Cyt	*P. patens*	168,012,416	71,002/5.96	80,637/6.37	3	54	2	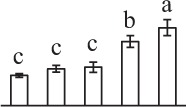
**PROTEIN SYNTHESIS (10)**										
83	Predicted protein, 40S ribosomal protein SA (RPSA)	Cyt	*P. patens*	168,017,628	31,539/4.72	43,804/4.58	7	101	2	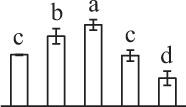
454	Hypothetical protein AMTR_s00087p00135370, containing cd08065 eukaryotic translation initiation factor 3^*^ (eIF3)	^#^Chl, Cyt, Mit, Nuc	*Amborella trichopoda*	548,842,775	44,710/5.17	39,587/4.64	5	82	2	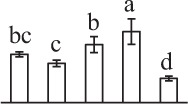
66	Hypothetical protein, eukaryotic initiation factor 4A^*^ (eIF4A)	Cyt	*P. patens*	168,026,095	47,119/5.46	48,999/5.32	25	162	11	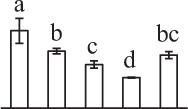
878	Eukaryotic initiation factor 4A (eIF4A)	Cyt	*Nicotiana. tabacum*	1,170,511	47,098/5.37	49,134/5.23	14	118	6	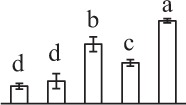
94	OSJNBa0020P07.3, elongation factor 2^*^ (EF2)	Cyt	*O. sativa* subsp. *japonica*	38,344,860	94,939/5.85	115,425/6.42	9	111	7	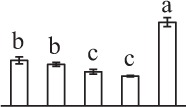
538	Hypothetical protein SELMODRAFT_411087, elongation factor 2^*^ (EF2)	Cyt	*S. moellendorffii*	302,773,640	94,568/6.00	114,195/6.33	5	167	5	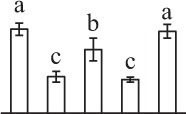
510	Hypothetical protein SELMODRAFT_143627, elongation factor G^*^ (EF-G)	^#^Chl, Mit	*S. moellendorffii*	302,765,284	75,483/5.31	119,422/6.41	6	75	4	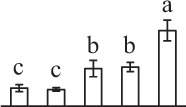
942	Elongation factor Tu (EF-Tu)	Mit	*Setaria italica*	514,812,465	48,530/5.99	44,724/6.18	16	63	8	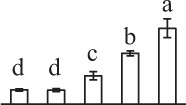
205	Uncharacterized protein LOC105056625, containing cd14275 elongation factor Ts domain^*^ (EF-Ts)	Chl	*Elaeis guineensis*	743,840,139	125,269/4.92	27,102/5.82	2	130	5	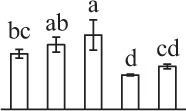
625	Tyrosine phosphorylated protein A (TypA)	Chl	*Suaeda salsa*	162,424,768	75,474/6.71	85,342/5.71	6	205	4	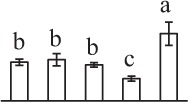
**PROTEIN FOLDING AND PROCESSING (8)**										
542	Hypothetical protein SELMODRAFT_440382, containing pfam00012 heat shock protein 70 domain^*^ (HSP70)	Cyt	*S. moellendorffii*	302,770,212	71,931/5.17	80,237/5.18	13	295	7	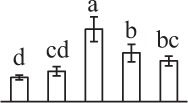
1002	Heat shock protein 70 (HSP70)	Cyt	*Populus trichocarpa*	224,098,390	71,620/5.14	80,105/5.21	16	135	8	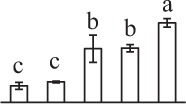
1566	Heat shock protein 70 (HSP70)	Cyt	*Petunia* × *hybrida*	20,559	71,137/5.07	106,146/6.34	4	92	2	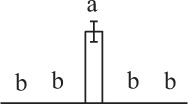
314	Heat shock protein 70 (HSP70)	Cyt	*Dactylis glomerata*	188,011,548	72,002/5.03	86,310/5.38	21	338	13	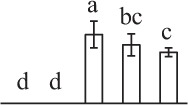
928	Heat shock protein 70 like protein (HSP70)	Cyt	*S. lycopersicum*	460,394,037	72,308/5.16	91,122/5.25	7	122	5	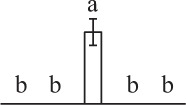
598	Predicted protein, containing pfam00183 heat shock protein 90 domain^*^ (HSP90)	Cyt	*P. patens*	168,034,606	79,652/4.93	87,379/5.25	5	89	3	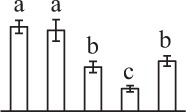
259	T-complex protein 1 subunit alpha (TCP1α)	Cyt	*A. thaliana*	135,535	59,477/5.93	66,881/6.22	8	246	4	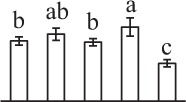
106	Hypothetical protein VITISV_000290, T-complex protein 1 subunit gamma^*^ (TCP1γ)	Cyt	*V. vinifera*	147,784,740	61,064/6.06	72,989/6.14	5	94	3	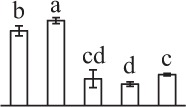
**PROTEIN DEGRADATION (8)**										
284	Alpha7 proteasome subunit (PSA7)	Cyt, Nuc	*N. tabacum*	14,594,925	27,466/6.11	25,449/5.20	8	79	2	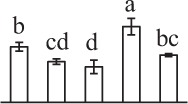
199	Zinc dependent protease (ZDP)	Chl	*Trifolium pratense*	84,468,286	74,746/5.82	73,106/5.66	12	106	7	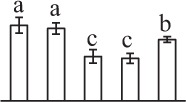
227	Zinc dependent protease (ZDP)	Chl	*T. pratense*	84,468,286	74,746/5.82	26,150/5.94	10	87	7	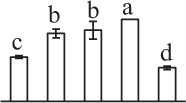
37	Zinc dependent protease (ZDP)	Chl	*T. pratense*	84,468,286	74,746/5.82	72,520/5.57	5	93	3	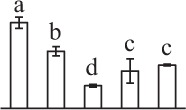
869	Zinc metalloprotease (ZMP)	Chl, Mit	*A. thaliana*	10,120,424	121,539/5.39	131,816/5.10	1	44	2	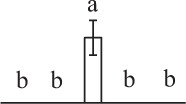
354	Predicted protein, containing pfam06480 FtsH extracellular domain^*^ (FtsH)	Chl	*Micromonas pusilla* CCMP1545	303,275,720	77,421/5.29	75,882/5.26	6	113	4	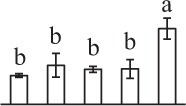
508	Predicted protein, ATP-dependent zinc metalloprotease FtsH^*^ (FtsH)	Chl	*P. patens*	168,001,910	68,933/5.23	108,174/5.50	3	75	2	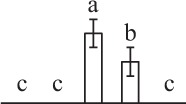
59	ATP-dependent Clp protease ATP-binding subunit ClpC (CLPC)	Chl	*A. thaliana*	9,758,239	103,616/6.36/	94,506/5.45/	9	415	9	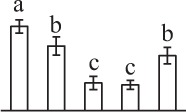
**STRESS AND DEFENSE (7)**										
414	Hypothetical protein MIMGU_mgv1a021611mg, containing cd03015 2-cys peroxiredoxin domain^*^ (Prx)	Chl, Cyt	*Erythranthe guttata*	604,334,612	21,153/4.98	21,812/4.68	17	132	4	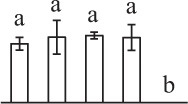
92	2-cys peroxiredoxin-like protein (Prx)	Chl, Cyt	*Hyacinthus orientalis*	47,027,073	21,956/4.93	22,930/5.24	12	58	3	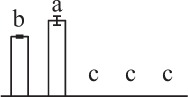
166	Ascorbate peroxidase (APX)	Cyt	*Eucalyptus grandis*	702,241,628	27,613/6.07	25,671/5.66	7	104	2	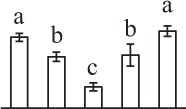
244	Dehydroascorbate reductase-like protein (DHAR)	Cyt	*S. tuberosum*	76,573,291	23,610/6.32	24,534/5.60	11	147	3	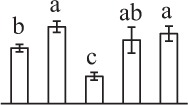
361	Dehydroascorbate reductase-like protein (DHAR)	Cyt	*S. tuberosum*	76,573,291	23,610/6.32	24,783/5.64	11	105	3	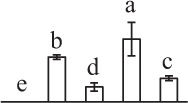
899	Dehydroascorbate reductase-like protein (DHAR)	Cyt	*S. tuberosum*	76,160,951	23,596/6.09	44,783/6.60	11	92	3	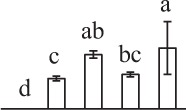
294	Chloroplast drought-induced stress protein of 32 kDa (CDSP32)	Chl, Cyt	*S. tuberosum*	2,582,822	33,779/8.07	31,436/6.26	8	87	3	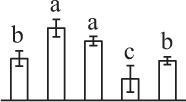

a *Assigned spot number as indicated in Figure 3*.

b The name and functional category of the proteins identified by ESI-Q-TOF and ESI-Q-Trap tandem mass spectrometry. Protein names marked with an asterisk (^*^) have been edited by us depending on searching against NCBI non-redundant protein database for functional domain. The abbreviations for the protein names are indicated in the bracket after protein names.

c *Protein subcellular localization predicted by softwares (YLoc, LocTree3, Plant-mPLoc, ngLOC, and TargetP). Only the consistent predictions from at least two tools were accepted as a confident result. Pounds (#) indicate prediction results were inconsistent among five tools. The subcellular localizations were predicted based on literature listed in Supplementary Table S4. Chl, chloroplast; Cyt, cytoplasm; Gol, Golgi apparatus; Mit, mitochondria; Nuc, nucleus; Pox, peroxisome*.

d *The plant species that the peptides matched from*.

e *Database accession number from NCBI non-redundant protein database*.

f,g *Theoretical (f) and experimental (g) molecular weight (Da) and pI of identified proteins. Theoretical values were retrieved from the protein database. Experimental values were calculated using ImageMaster 2D version 5.0*.

h *The amino acid sequence coverage for the identified proteins*.

i *The Mascot score obtained after searching against the NCBI non-redundant protein database*.

j *The number of matched peptides for each protein*.

k *The mean values of protein spot volumes relative to total volume of all the spots. Five spore germination stages, MS, mature spores; RS, rehydrated spores, DCS, double-celled spores, GS, germinated spores; and SPC, spores with protonemal cells were performed. Error bar indicates ± standard deviation (SD). Letters indicate statistically significant differences (p <0.05) among five stages of spore germination as determined by One-Way ANOVA*.

**Figure 4:**
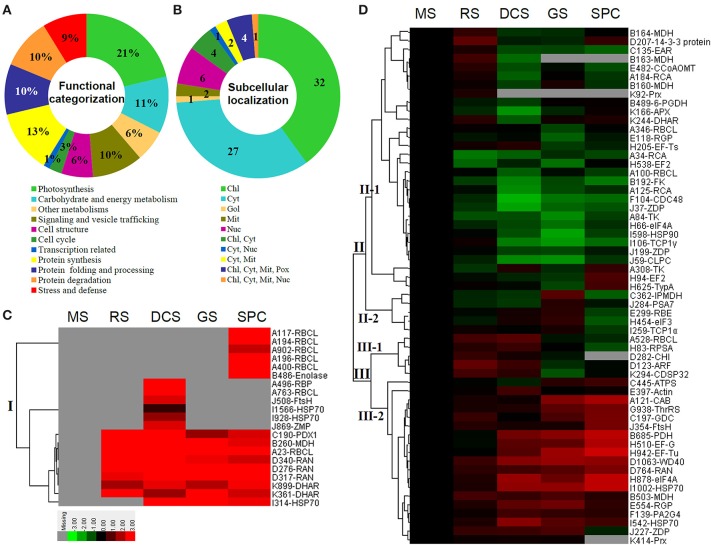
**Functional categorization, subcellular localization, and hierarchical clustering of differentially abundant proteins upon** ***E. arvense*** **spore germination. (A)** A total of 80 proteins were classified into 11 functional categories. The percentage of proteins in different functional categories are shown in the pie. **(B)** Subcellular localization categories of proteins predicted based on softwares and literature. The numbers of proteins with different locations are shown in the pie. Chl, chloroplast; Cyt, cytoplasm; Gol, Golgi apparatus; Mit, mitochondria; Nuc, nucleus; Pox, peroxisome. **(C,D)** Dendrogram of 80 differentially abundant proteins obtained by hierarchical clustering analysis. The columns represent different spore germination stages, including MS, mature spores; RS, rehydrated spores; DCS, double-celled spores; GS, germinated spores; SPC, spores with protonemal cells. The rows represent individual proteins. Three main clusters (I–III) and subclusters of II and III (II-1, II-2, III-1, and III-2) are shown on the left side. Functional categories indicated by capital letters, spot numbers, and protein name abbreviations are listed on the right side. The scale bar indicates log (base 2) transformed protein abundance ratios ranging from -13.0 to 3.0. The ratio was calculated as protein abundance at MS stage divided by abundance each stage, respectively. The increased and decreased proteins are represented in red and green, respectively. The color intensity increases with increasing abundant differences. Undetected proteins are indicated in gray. Abbreviations for functional categories: A, Photosynthesis; B, Carbohydrate and energy metabolism; C, Other metabolisms; D, Signaling and vesicle trafficking; E, Cell structure; F, Cell cycle; G, Transcription related; H, Protein synthesis; I, Protein folding and processing; J, Protein degradation; K, Stress and defense. Detailed information on protein names and abbreviations can be found in Table 1.

The 80 DAPs were classified into eleven groups, including photosynthesis (17), carbohydrate and energy metabolism (9), other metabolisms (5), signaling and vesicle trafficking (8), cell structure (5), cell cycle (2), transcription related (1), protein synthesis (10), protein folding and processing (8), protein degradation (8), stress and defense (7) (Table 1). Among them, proteins involved in photosynthesis (21%) and protein synthesis (13%) were over-represented (Figure 4A), indicating active photosynthesis and *de novo* protein synthesis are pivotal for the germinating chloropyllous spores.

Interestingly, the 80 DAPs represented 48 unique proteins. Thirteen proteins (16.3%) had multi-proteoforms, which were mainly involved in photosynthesis, tricarboxylic acid (TCA) cycle, vesicle trafficking, protein synthesis, folding and turnover, as well as reactive oxygen species (ROS) scavenging (Table 1, Supplementary Table S3). These proteoforms might be generated from alternative splicing and various post-translational modifications.

The subcellular localization of the 80 DAPs was predicted based on five internet tools (i.e., YLoc, LocTree3, ngLOC, TargetP, and Plant-mPLoc) and literature. In total, 32 DAPs were predicted to be localized in chloroplast, 27 in cytoplasm, one in Golgi apparatus, two in mitochondria, and six in nucleus. Besides, seven proteins were predicted to be localized in two organelles, and five proteins in four organelles (Figure 4B, Supplementary Table S4).

To better understand the protein expression characteristics during the five stages, hierarchical clustering analysis was applied to the 80 proteins, which revealed three main clusters. Cluster I contained 21 proteins, which were not expressed in MS, but induced in other stages of germination. They were involved in photosynthesis, glycolysis, protein folding and turnover, signal transduction, and ROS scavenging, implying these pathways were specially induced in certain germination stages (Figure 4C). Cluster II included 35 proteins that were mainly decreased during spore germination. These proteins were divided into two subclusters. Subcluster II-1 contained the proteins mainly decreased during germination, and subcluster II-2 included proteins induced at the stage of GS but obviously reduced in SPC. The proteins in cluster II covered ten function categories, in addition to category of transcription (Figure 4D). The rest 24 proteins were grouped into cluster III, representing increased proteins during spore germination. Cluster III contained two subclusters. Subcluster III-1 contained five proteins induced in stages of RS and DCS, but reduced in GS and SPC, and subcluster III-2 included 19 proteins significantly increased at all stages of germination (Figure 4D).

### Photosynthesis and reserve mobilization are active in the germinating spores

Mature chlorophyllous spores of *Equisetum* species contain chloroplasts and water, making them metabolically active and short-lived. After released from sporangium, the green spores can survive desiccation for less than 2 weeks (Lebkuecher, 1997). It has been found that the *E. hyemale* spores can tolerate desiccation of 2% relative humidity for 24 h. Upon rehydration, they can rapidly regain photosynthetic competence (Lebkuecher, 1997). In this study, we found the viability of spores from *E*. *arvense* lasted less than 3 weeks, and the fresh spores germinated in 32 h (Figure 1). The extremely short viability and rapid germination were mainly due to active photosynthesis and respiration in the chlorophyllous spores. This is different from non-green spores with long dormancy and viability (Lloyd and Klekowski, 1970). Upon germination, chlorophyllous spores also exhibited features distinct from the non-green spores. In our proteomic results, we found that 17 DAPs were photosynthesis-related proteins, including a chlorophyll a/b-binding protein, 10 proteoforms of ribulose-1,5-bisphosphate carboxylase/oxygenase (RuBisCO) large subunit, a RuBisCO large subunit binding protein, three proteoforms of RuBisCO activase, and two proteoforms of chloroplast transketolase (Table 1). The increased chlorophyll a/b-binding protein indicated that the photosystem II was enhanced upon spore germination. The multiple proteoforms of RuBisCO large subunit, RuBisCO activase, and transketolase changed in abundance possibly due to protein phosphorylation (Guitton and Mache, 1987). This implies that the carbon assimilation is active in germinating spores. Importantly, we also found nine enzymes involved in carbohydrate and energy metabolism. They were six TCA cycle enzymes (i.e., a pyruvate dehydrogenase and five malate dehydrogenases), an enolase involved in glycolysis, a cytosolic 6-phosphogluconate dehydrogenase (an enzyme in pentose phosphate pathway), and a fructokinase in charge of sucrose and fructose metabolism. All these enzymes, taking up 32% of DAPs, were considered as key enzymes for carbon and energy supplies during spore germination. Their variations also indicate that the heterotrophic metabolism is crucial for chlorophyllous spore germination.

We also found several proteins involved in fatty acid synthesis, amino acid metabolism, sulfur assimilation, and secondary metabolism (Table 1). Among them, enoyl-acyl carrier protein reductase is a key enzyme of the type II fatty acid synthesis system in plastids. Enoyl-acyl carrier protein reductase was proved to be important for fatty acid deposition in developing seeds (de Boer et al., 1999) and pollen grains (Poghosyan et al., 2005). In our results, enoyl-acyl carrier protein reductase was decreased at the stages of DCS, GS and SPC during germination. This indicates that fatty acid synthesis is reduced upon spore germination, which is consistent with the notion that mobilization of storage lipids has been triggered for energy supply in germinating spores (DeMaggio and Stetler, 1985). Besides, we found three increased enzymes at certain stages involved in amino acid metabolism, including glycine decarboxylase, 3-isopropylmalate dehydrogenase (IPMDH), and ATP-sulfurylase. Glycine decarboxylase participates in glycine, serine and threonine metabolism, and was found to be abundant in mitochondria of C3 leaves that functions in photorespiratory carbon recovery (Timm et al., 2012). Whether glycine decarboxylase is involved in photorespiratory in germinating fern spores needs to be further investigated. IPMDH catalyzes the oxidative decarboxylation of 3-isopropylmalate in leucine biosynthesis, and was proved to be essential for Arabidopsis pollen development (He et al., 2011). In our results, IPMDH was obviously induced in the stage of GS, suggesting that IPMDH is also pivotal for spore germination. Interestingly, ATP-sulfurylase acts as the metabolic entry point into the sulfur assimilation pathway. It was found that the increase of ATP-sulfurylase level during soybean seed development could lead to an increase in the availability of sulfur amino acids (Phartiyal et al., 2006). The induced ATP-sulfurylase in GS and SPC may facilitate the synthesis of sulfur rich amino acids for spore germination. Additionally, pyridoxal biosynthesis protein (PDX) was specially expressed in germinating spores, but not found in MS. PDX family in plants is primarily known for its role in vitamin B6 biosynthesis. Recently, PDX1.2 was proved to be critically required for hypocotyl elongation and primary root growth (Leuendorf et al., 2014). The increase of PDX in germinating spores implies that it probably functions in rhizoid elongation.

### Signaling and vesical trafficking are important for spore germination

The critical roles of hormone signaling and vesical trafficking in germinating seeds and pollen grains have been well-studied (Ellis and Turner, 2002; Dai et al., 2007a), but little information is available for fern spore germination. In this study, we found some novel factors in signal transduction and vesical trafficking upon fern spore germination, including chalcone isomerase, WD40, 14-3-3 protein, GTP-binding nuclear protein Ran (RAN), and ADP-ribosylation factor (ARF) (Table 1). Among them, chalcone isomerase involved in flavonoid biosynthesis was increased at the stage of RS. Flavonoid participates in auxin signaling and facilitates pollen-tube growth (Falcone Ferreyra et al., 2012). In maize (*Zea mays*) and *Petunia hybrida* mutants, the flavonoid-deficient pollen failed to produce a functional pollen tube (Mo et al., 1992). The increase of chalcone isomerase in spores implies that flavonoids may function in fern spore germination as well. Interestingly, flavonoid biosynthesis is regulated by conserved WD40 domain-contained transcription factors (Falcone Ferreyra et al., 2012). Members of WD40 protein superfamily are known as key regulators of multi-cellular processes (e.g., cell division, light signaling, protein trafficking, cytoskeleton dynamics, nuclear export, RNA processing, chromatin modification, and transcriptional mechanism), acting as scaffolding molecules assisting proper activity of other proteins (Stirnimann et al., 2010). Moreover, some WD40 proteins have been found to be crucial for Arabidopsis seed germination (Gachomo et al., 2014) and pollen viability in flax (*Linum usitatissimum* L.) (Kumar et al., 2013). In our results, the nucleus-localized WD40 was induced in horsetail germinating spores, indicating its regulatory function for spore germination and rhizoid tip-growth. Similarly, 14-3-3 protein family is also a highly conserved eukaryotic proteins with multiple molecular and cellular functions by binding to phosphorylated client proteins to modulate their function (Denison et al., 2011). In lily (*Lilium longiflorum*) pollen, 14-3-3 protein was involved in the regulation of plasma membrane H^+^-ATPase via modulation of its activity, which is essential for germination and tube elongation (Pertl et al., 2010). In this study, we found a 14-3-3 protein was induced in the stages of RS and SPC, indicating it would play roles in cell nuclear migration and rhizoid tip-growth. Importantly, we also found four proteoforms of RAN were significantly increased in germinating spores. RAN, a primarily nucleus-localized small GTPase, is essential for nuclear transport and assembly, mRNA processing, and cell cycle (Ciciarello et al., 2007). It was reported that overexpression of *RAN1* in rice (*Oryza sativa*) and Arabidopsis altered the mitotic progress and sensitivity to auxin (Wang et al., 2006). The multi-proteoforms of RAN induced in germinating horsetail spores were probably due to the different phosphorylation levels, implying their probable key roles in cell division and polar growth during spore germination. Besides of RAN, we found another important small GTPase, ARF, was induced at the stages of RS and DCS. ARF contributes to the regulation of multiple trafficking routes with respect to Golgi organization, endocytic cycling, cell polarity and cytokinesis (Yorimitsu et al., 2014). It was found that ARF played essential roles for endosomal recycling during Arabidopsis pollen and root hair polarized tip growth (Richter et al., 2011). The induced ARF in horsetail spores meets the specific requirement of early-secretory and polar vesical recycling to facilitate rhizoids tip growth.

### Cytoskeleton and cell wall dynamics are necessary for spore germination

Actin cytoskeleton directs the flow of vesicles to the apical domain, where they fuse with the plasma membrane and contribute their contents to the expanding cell wall (Hepler et al., 2013). The local changes in contents and viscosity of the apical wall control the local expansion rate and cell elongation. Precise mechanisms in the organization of actin cytoskeleton and cell wall dynamics have been well-studied in growing pollen tubes (Hepler et al., 2013; Qu et al., 2015), but the mechanisms remain to be further elucidated in fern spores. Here we found the levels of actin, reversibly glycosylated polypeptide, rhamnose biosynthetic enzyme 1, and caffeoyl-CoA O-methyltransferase (CCoAOMT) were altered during horsetail spore germination. Actin is known to be the main content of actin cytoskeleton, and the other three enzymes are essential for cell wall modulation. Reversibly glycosylated polypeptide is implicated in polysaccharide biosynthesis (Langeveld et al., 2002), and may function in cell wall construction in pollen from rice and *Picea meyeri* (Dai et al., 2006; Chen et al., 2009). Besides, rhamnose biosynthetic enzyme catalyzes the synthesis of L-rhamnose, an important constituent of pectic polysaccharides in cell wall of pollen tube (Yue et al., 2014). In addition, CCoAOMT has a proven role in lignin monomer biosynthesis, which is crucial for pollen wall development (Arnaud et al., 2012). In our results, rhamnose biosynthetic enzyme was increased in GS and decreased in SPC, CCoAOMT was induced in RS and reduced in the stages of DCS and SPC, and two proteoforms reversibly glycosylated polypeptide displayed different changes. All these alterations would modulate the biosynthesis of cell wall components (e.g., polysaccharide, rhamnose, and lignin), leading to the dynamics of rhizoid cell wall rigidity for sustained polarized growth.

### Rapid protein synthesis, processing, and turnover are essential for fern spore germination

The germinating pollen and fern spores exhibit quick switches from metabolic quiescent state to active state. The substance and energy supply for rapid cell division and polar tip-growth need to be triggered in a short time period. Although it has been found that mature pollen grains have pre-synthesized mRNA and proteins for germination and tube growth (Taylor and Hepler, 1997; Dai et al., 2006), *de novo* protein synthesis is necessary for pollen tube elongation (Dai et al., 2007a,b). For fern species, the germination of green spores from *O. sensibilis* and non-green spores from *A. phyllitidis, Marsilea vestita, Pteridium aquilinum*, and *Pteris vittata* were not inhibited by actinomycin D (Raghavan, 1977, 1991, 1992; Kuligowski et al., 1991). In horsetail spores, similar metabolic features were discovered from our proteomics data. We found eleven DAPs involved in transcription and protein synthesis, including a threonyl-tRNA synthetase, a 40S ribosomal protein SA, three eukaryotic translation initiation factors (eIFs) (a eIF3 and two eIF4A), five elongation factors (EFs) (two proteoforms of EF2, a EF-G, a EF-Tu, and a EF-Ts), and a protein translation-related GTP-binding protein tyrosine phosphorylated protein A (TypA) (Table 1). The increases of threonyl-tRNA synthetase in GS and SPC and increases of 40S ribosomal protein SA in RS and DCS indicate that the protein synthesis machinery is enhanced during spore germination, while the changes in multi-proteoforms of eIF and EF imply that certain specific protein synthesis is regulated in diverse modes in different stages of germinating spores. It is interesting to note that these proteins are localized in cytoplasm, mitochondria, and chloroplast, respectively (Table 1). This indicates that not only the synthesis of nuclear gene-encoding proteins are necessary, but also the protein synthesis machineries in chloroplast and mitochondria are all triggered for the active metabolism in germinating spores.

Molecular chaperones not only control house-keeping processes, but also regulate protein functional folding and assembly which are necessary for activating/inhibiting various signaling pathways. In horsetail germinating spores, we found eight chaperones, including five proteoforms of heat shock protein 70 (HSP70), HSP90, T-complex protein 1 (TCP1) subunit alpha, and TCP1 subunit gamma. Among these HSP members, HSP70 was found to bind microtubules and interact with kinesin in tobacco (*Nicotiana tabacum*) pollen tubes (Parrotta et al., 2013). Besides, Arabidopsis HSP90 was found to be active in mature and germinating pollen grains (Prasinos et al., 2005). Moreover, TCP1 plays a pivotal role in the folding and assembly of cytoskeleton proteins as an individual or complex with other subunits (Sternlicht et al., 1993; Bhaskar et al., 2012). The variations of chaperones in spores highlight that protein processing is important for diverse processes (e.g., photosynthesis, cytoskeleton, protein turnover, and various metabolisms) upon horsetail spore germination.

In addition to folding and assembly, active protein turnover occurred in germinating horsetail spores, which was reflected by the changes of eight degradation-related proteins (Table 1). These proteins included an alpha 7 proteasome subunit, three proteoforms of zinc dependent protease, a zinc metalloprotease, two proteoforms of FtsH protease, and an ATP-dependent Clp protease (Table 1). Among them, all the zinc dependent proteases, zinc metalloprotease, and FtsH are all ATP-dependent metalloproteases in chloroplasts, which play a major role in assembly and maintenance of the plastidic membrane system. The Clp protease system plays essential role in plastid development through selective removal of miss-folded, aggregated (Nishimura and van Wijk, 2014), or unwanted proteins. All these imply that active protein degradation and turnover in chloropyllous spores are crucial for spore germination.

### ROS homeostasis is crucial for spore germination

In the tip-growing pollen tubes and root hairs, ROS act as regulators in diverse signal and metabolic pathways, modulating kinase cascades, ion channels, cell wall properties, nitric oxide levels, and G-protein activities (Wilson et al., 2008; Swanson and Gilroy, 2010). Interestingly, in fern spores, nitric oxide was shown to be a positive regulator for tip growth (Bushart and Roux, 2007). However, the ROS function in fern spores is poorly understood. Our proteomics results revealed that seven proteins in germinating spores function as ROS scavengers, including two proteoforms of 2-cys peroxiredoxin (Prx), a chloroplast drought-induced stress protein of 32 kDa (CDSP32), ascorbate peroxidase (APX), and three proteoforms of dehydroascorbate reductase (DHAR). The proteoforms of Prx, CDSP32, DHAR, and APX are predicted to be localized in chloroplasts and/or cytoplasm in horsetail spores (Table 1). CDSP32 is composed of two thioredoxin (Trx) modules and has been found to be involved in the protection of the photosynthetic apparatus against oxidative damage (Broin et al., 2002). PrxR/Trx pathway is a central antioxidant defense system in plants, in which PrxRs employ a thiol-based catalytic mechanism to reduce H_2_O_2_ and is regenerated using Trxs as electron donors. APX and DHAR are the key members in glutathione-ascorbate cycle. In this cycle, H_2_O_2_ is reduced to water by APX using ascorbate as the electron donor. The oxidized ascorbate is still a radical, which can be converted into dehydroascorbate spontaneously or reduced to ascorbate by monodehydroascorbate reductase. Dehydroascorbate is then reduced to ascorbate by DHAR at the expense of glutathione, yielding oxidized glutathione. Finally, oxidized glutathione is reduced by glutathione reductase using NADPH as electron donor (Horling et al., 2003). Previous proteomics studies have revealed that the abundances of APX and Trx were changed in germinating pollen grains from *O. sativa* (Dai et al., 2007a), Arabidopsis (Ge et al., 2011), *Brassica napus* (Sheoran et al., 2009), *P. meyeri* (Chen et al., 2009), *Picea wilsonii* (Chen et al., 2012), and *Pinus strobus* (Fernando, 2005). This indicated that PrxR/Trx pathway and glutathione-ascorbate cycle were employed in germinating pollen grains. Thus, our results indicate chlorophyllous fern spores with active photosynthesis and respiration modulate ROS homeostasis by these enzymes during germination.

### Prediction of protein-protein interaction upon spore germination

**Figure 5:**
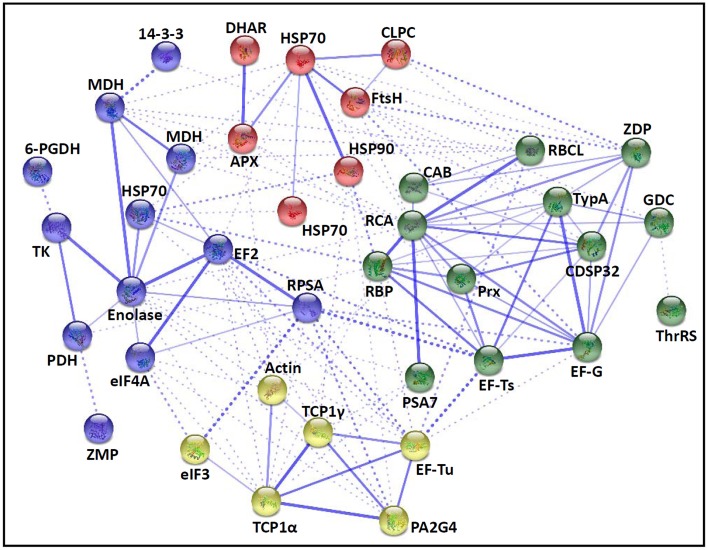
**The protein-protein interaction (PPI) network of proteins in** ***E. arvense*** **spores based on STRING analysis**. A total of 63 differentially abundant proteins represented by 38 unique homologous proteins from Arabidopsis are shown in PPI network. Nodes in different colors belong to four main groups. The PPI network is shown in the confidence view generated by STRING database. Strong associations are represented by thicker lines. Detailed information on protein names and abbreviations can be found in Table 1.

The fern spore germination is a fine-tuned process regulated by temporal and spatial expression and interaction of a number of genes/proteins. To discover the relationship of DAPs during horsetail spore germination, the protein-protein interaction networks were generated by the web-tool STRING 9.1 (http://string-db.org). The DAPs homologs in Arabidopsis were found by sequence BLASTing in TAIR database (http://www.arabidopsis.org/Blast/index.jsp) (Supplementary Table S5), and then the homologs were subjected to the molecular interaction tool of STRING 9.1 for creation of proteome-scale interaction network. Among all the 80 DAPs, 63 proteins identified in germinating spores and represented by 38 unique homologous proteins from Arabidopsis, were depicted in the STRING database (Figure 5), according to the information from the published literature, genome analysis based on domain fusion, phylogenetic profiling/homology, gene neighborhood, co-occurrence, co-expression, and other experimental evidence (Supplementary Figure S4). Four functional modules are apparently illuminated in the network, which form tightly connected clusters (Figure 5). In the protein networks, stronger associations are represented by thicker lines (Figure 5). In Module 1 (green nodes), photosynthetic proteins (chlorophyll a/b-binding protein, RuBisCO large subunit, RuBisCO activase, and RuBisCO large subunit binding protein), three members of chloroplastic protein synthesis machine (EF-Ts, EF-G, and TypA), chloroplast-located Prx/Trx and zinc dependent protease, as well as a mitochondria-located glycine decarboxylase appeared linked closely. This implies that photosynthesis, photorespiration, chloroplastic protein synthesis and turnover, as well as ROS scavenging are active and cooperated closely in horsetail chloropyllous spores. Besides, multiple metabolic enzymes (e.g., two malate dehydrogenases, enolase, transketolase, pyruvate dehydrogenase, and 6-phosphogluconate dehydrogenase), cytoplastic members of protein synthesis machine (40S ribosomal protein SA, eIF4A, EF2), a molecular chaperone (HSP70), a protease (zinc metalloprotease), and 14-3-3 protein were assigned in Module 2 (blue nodes). These linked proteins show that diverse metabolic pathways (e.g., TCA cycle, glycolysis, pentose phosphate pathway, and Calvin cycle) formed a synergistic system for carbon and energy supplies during germination. Moreover, these metabolic activities were controlled by the levels and activities of key enzymes that were modulated through protein synthesis, folding, and turnover, while 14-3-3 proteins acted as a crucial regulator of these metabolic processes (e.g., TCA cycle) (Diaz et al., 2011). Interestingly, actin is linked with TCP1, proliferation-associated protein 2G4, and two members of protein synthesis machine (eIF3 and EF-Tu) in Module 3 (yellow nodes), indicating the synthesis and processing of cytoskeletal proteins are pivotal for the rapid cell division and cell cycle upon spore germination. Furthermore, proteins involved in protein folding (HSP70 and HSP90) and degradation (FtsH and ATP-dependent Clp protease), as well as ROS scavengers (APX and DHAR) were fitted into Model 4 (red nodes). This indicates that the protein conformational changes determine their fates and are regulated by ROS homeostasis in germinating spores.

## Conclusion and remarks

Although the molecular mechanism of pollen germination has been well-studied as a model for cell polar growth, our knowledge of fern chlorophyllous spore germination is lacking. In this proteomics study, we found some pollen homologous proteins and several novel components are pivotal for fern spore germination. The dynamics of photosynthesis, TCA cycle, glycolysis, and pentose phosphate pathway, as well as the variations of reserve mobilization pathways (fatty acid synthesis, amino acid metabolism, sulfur assimilation, and secondary metabolism) indicate that both heterotrophic and autotrophic metabolisms are triggered in chlorophyllous spores, which is obviously distinct with non-green spores and pollen grains. Besides, a number of proteins are suspected to be necessary for the cell nuclear migration, cytoskeleton dynamics, and cell wall modulation during fern spore germination. Importantly, the protein synthesis machines, protein processing, and proteasome-dependent protein degradation in cytoplasm and chloroplasts are active for the rapid protein synthesis and turnover. In addition, several members in ROS signaling and G protein-involved vesical trafficking are crucial for polar rhizoid growth. All these provide invaluable information, however, further validation and characterization of these proteins in a model system (i.e., *C. richardii*), as well as the post-translational modification analysis are still necessary for ultimately discovering protein functions and interactions toward understanding of the underlying sophisticated cellular and molecular processes in fern germinating spores.

### Conflict of interest statement

The authors declare that the research was conducted in the absence of any commercial or financial relationships that could be construed as a potential conflict of interest.

## Abbreviations

APX: ascorbate peroxidase
ARF: ADP-ribosylation factor
CCoAOMT: caffeoyl-CoA O-methyltransferase
CDSP32: chloroplast drought-induced stress protein of 32 kDa
DAP: differentially abundant protein
DCS: double-celled spores
DHAR: dehydroascorbate reductase
EF: elongation factor
eIF: eukaryotic translation initiation factor
GO: Gene Ontology
GS: germinated spores
HAI: hours after illumination
HSP: heat shock protein
IPMDH: 3-isopropylmalate dehydrogenase
MS: mature spores
PDX: pyridoxal biosynthesis protein
Prx: peroxiredoxin
RAN: GTP-binding nuclear protein Ran
ROS: reactive oxygen species
RS: rehydrated spores
RuBisCO: ribulose-1,5-bisphosphate carboxylase/oxygenase
SPC: spores with protonemal cells
TCA: tricarboxylic acid
TCP: T-complex protein
Trx: thioredoxin
TypA: tyrosine phosphorylated protein A.
