# Supplementary figures and images for: Cytological and proteomic analyses of horsetail (Equisetum arvense L.) spore germination
Source: Front Plant Sci. 2015 Jun 17;6:441. doi: 10.3389/fpls.2015.00441 (PMC4469821; doi:10.3389/fpls.2015.00441)

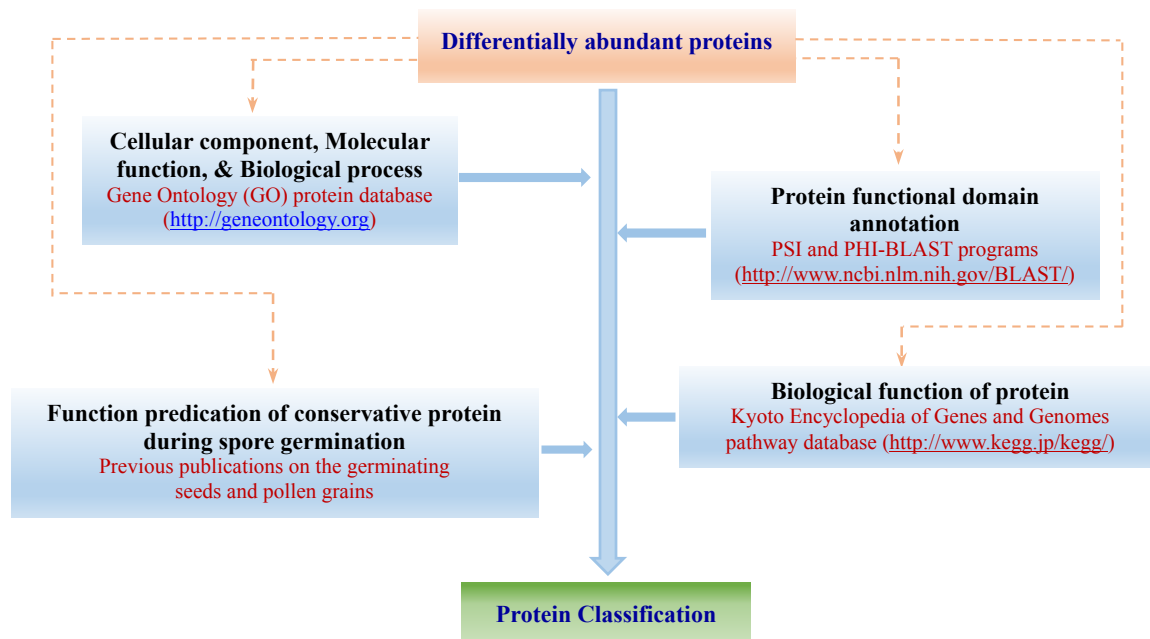

**Supplementary Figure S1. Workflow of protein function classification.**

Supplement: Supplementary file 1 [file Image1.PDF]
