# Supplementary material for: Cytological and proteomic analyses of horsetail (Equisetum arvense L.) spore germination
Source: Front Plant Sci. 2015 Jun 17;6:441. doi: 10.3389/fpls.2015.00441 (PMC4469821; doi:10.3389/fpls.2015.00441)
Supplement: Supplementary file 2 [file Image2.PDF]

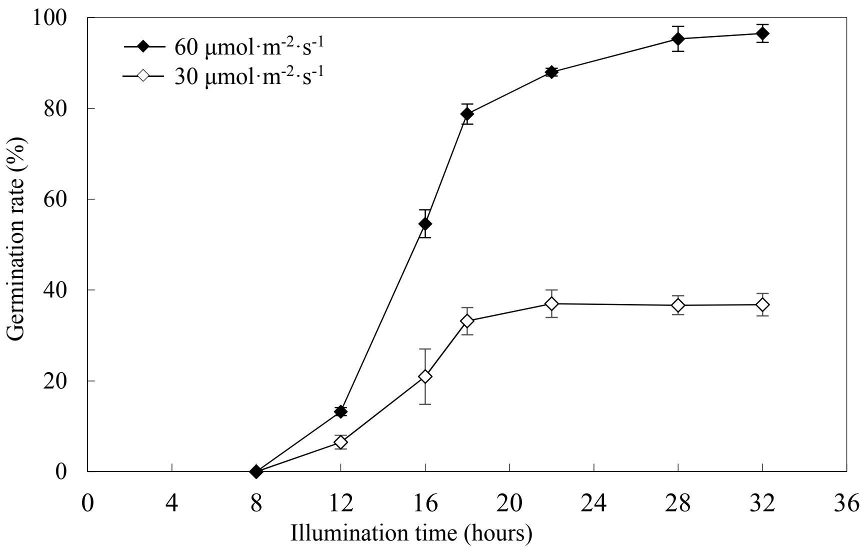

Supplementary Figure S2. Effects of illumination on the spore germination of *E. arvense*. *E. arvense* spores were cultured on Knop's medium under two different illumination levels (60 µmol·m^{-2}·s^{-1} and 30 µmol·m^{-2}·s^{-1}). The spore germination rate was defined as the ratio of germinated spore number to total spore number. The calculation of spore germination rate was started from 8 hours after illumination when the double-celled spores were formed. Filled diamonds represent spore germination rate under light quality of 60 µmol·m^{-2}·s^{-1}, and open diamonds represent spore germination rate under light quality of 30 µmol·m^{-2}·s^{-1}. Error bars indicate ± standard deviation.
