# Supplementary material for: Cytological and proteomic analyses of horsetail (Equisetum arvense L.) spore germination
Source: Front Plant Sci. 2015 Jun 17;6:441. doi: 10.3389/fpls.2015.00441 (PMC4469821; doi:10.3389/fpls.2015.00441)
Supplement: Supplementary file 3 [file Image3.PDF]

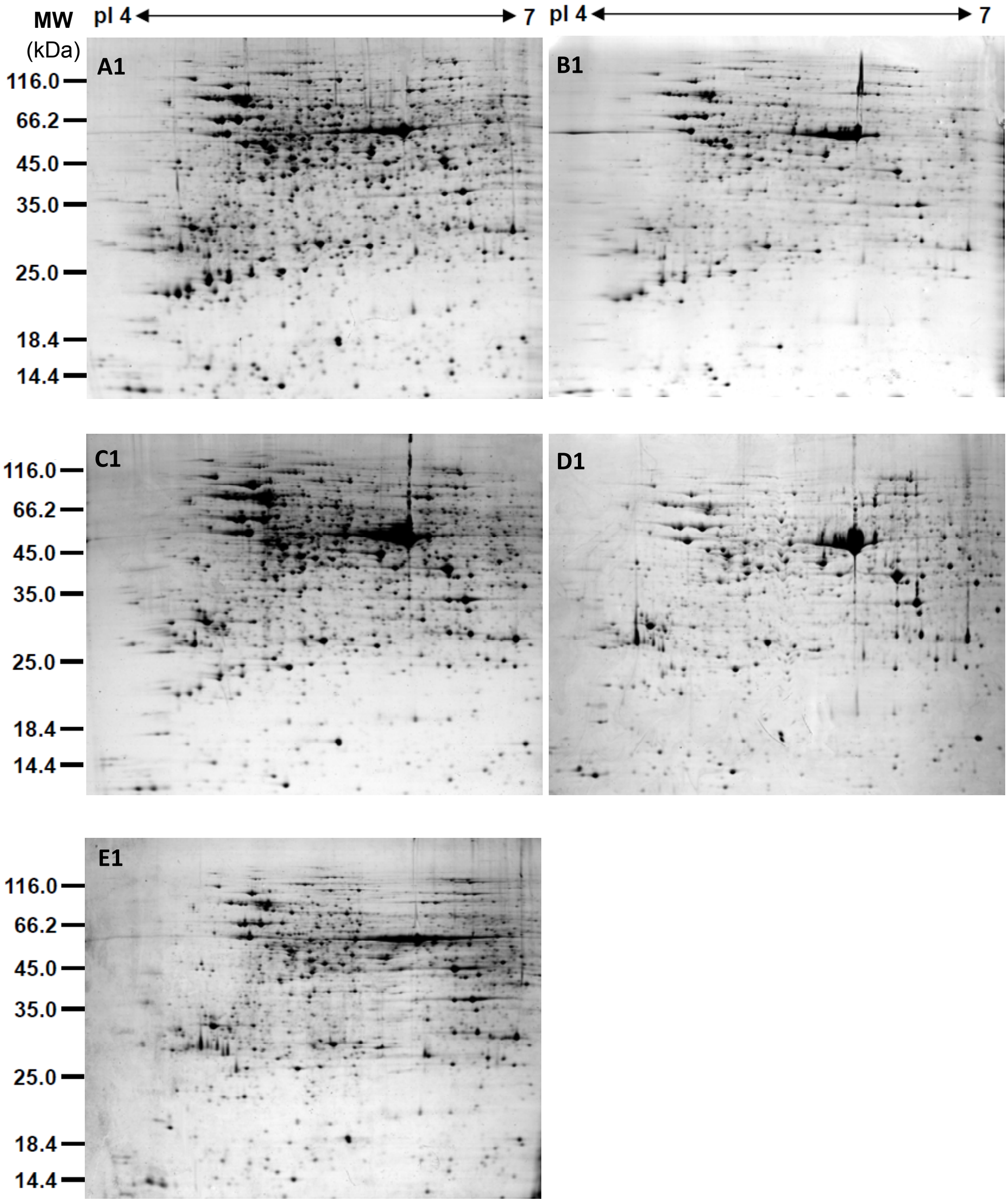
MW
(kDa)
pI 4
7
pI 4
7
A1
B1
C1
D1
E1
116.0
66.2
45.0
35.0
25.0
18.4
14.4

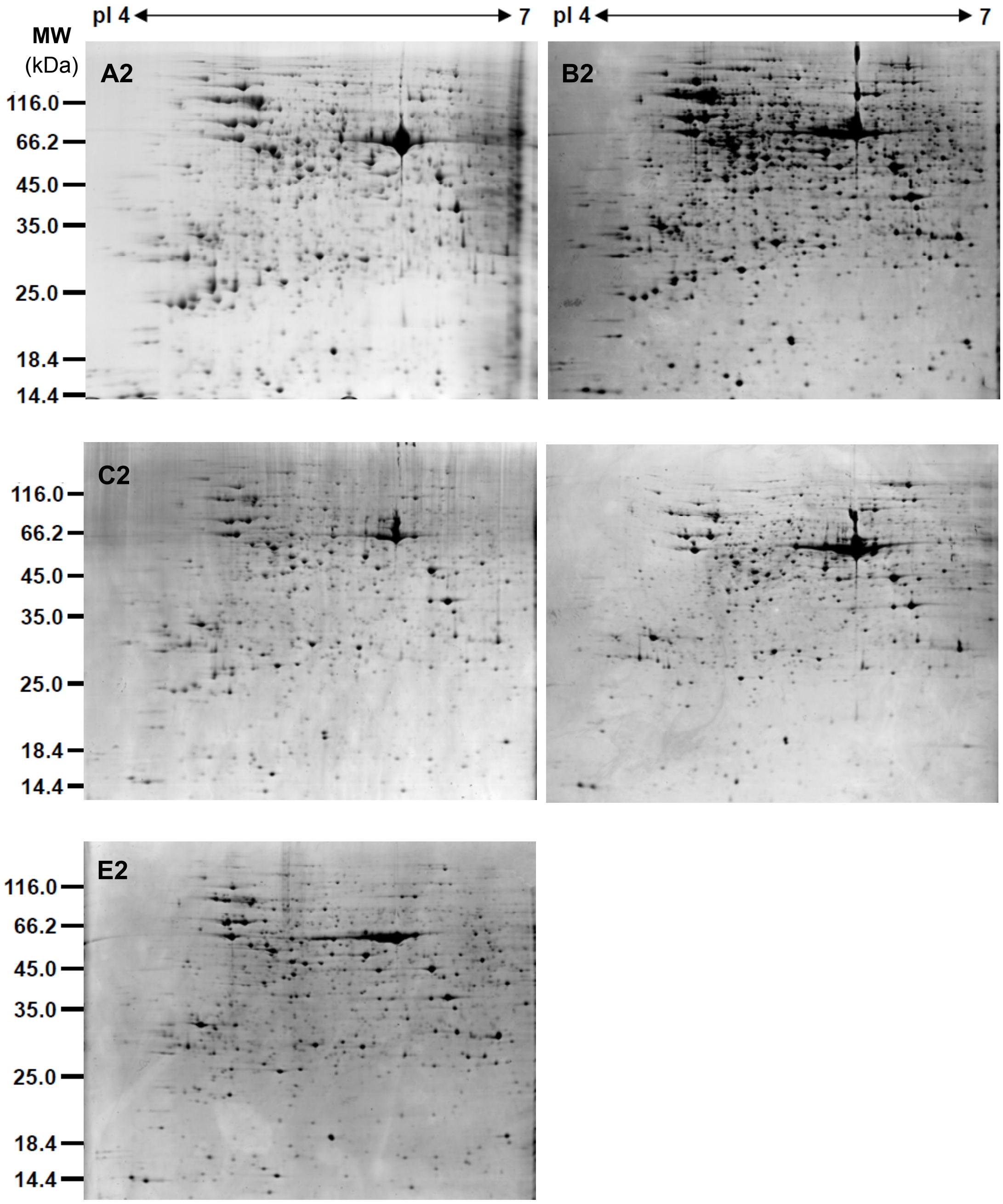

pI 4
7
pI 4
7
MW
(kDa)
A2
B2
C2
E2
116.0
66.2
45.0
35.0
25.0
18.4
14.4

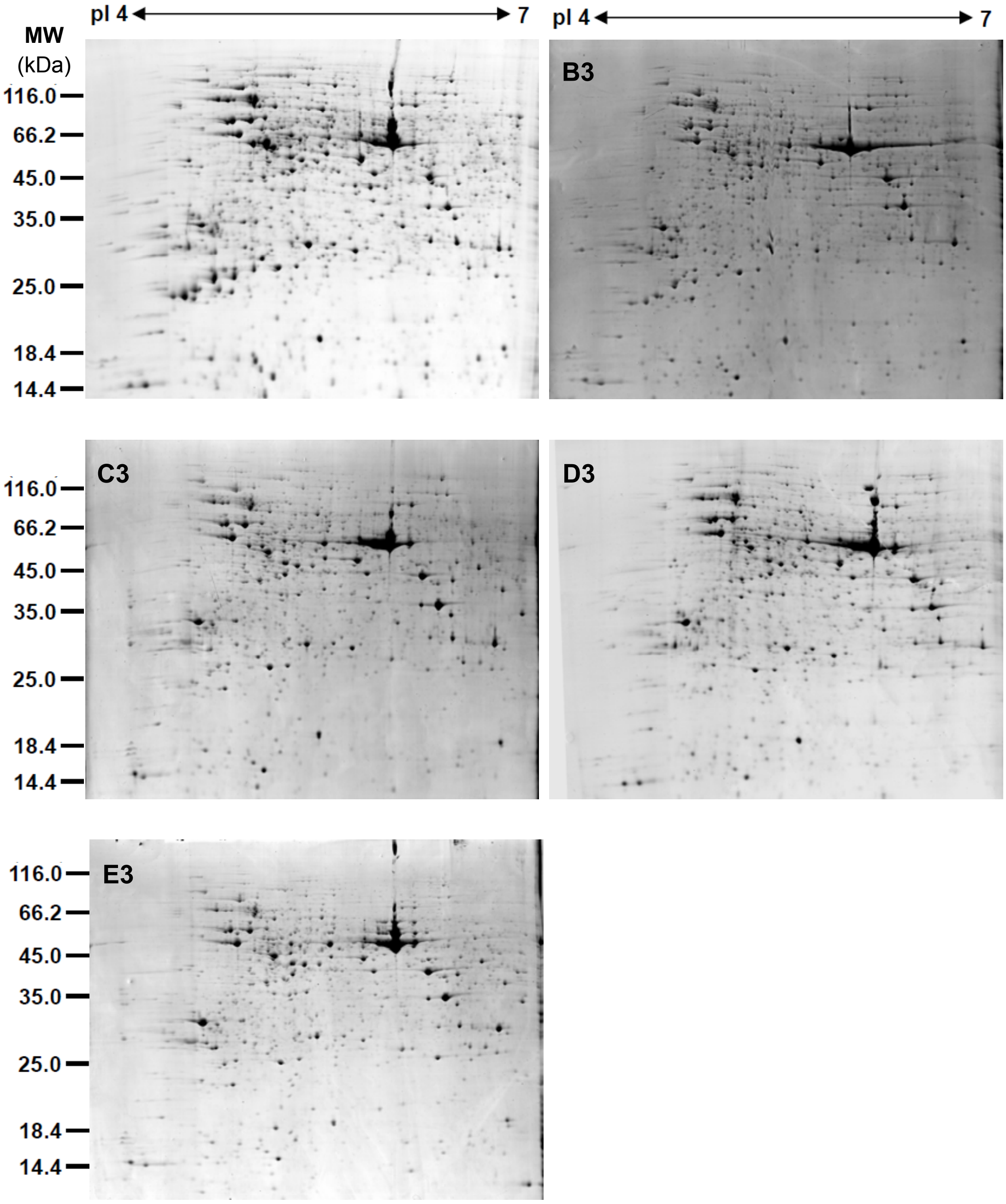

Supplementary Figure S3. Three biological replicates of 2-DE images of proteins in various germination stages from *E. arvense* spores. (A1-A3) Mature spores. **(B1-B3)** Rehydrated spores. **(C1-C3)** Double-celled spores. **(D1-D3)** Germinated spores. **(E)** Spores with protonemal cells. Proteins were separated on 24 cm IPG strips (pH 4-7 linear gradient) using IEF in the first dimension, followed by 12.5% SDS-PAGE gels in the second dimension. The 2-DE gel was stained with Coomassie Brilliant Blue. Molecular weight (MW) in kDa and pI of proteins are indicated on the left and top of the gels, respectively.
