# Supplementary material for: Cytological and proteomic analyses of horsetail (Equisetum arvense L.) spore germination
Source: Front Plant Sci. 2015 Jun 17;6:441. doi: 10.3389/fpls.2015.00441 (PMC4469821; doi:10.3389/fpls.2015.00441)
Supplement: Supplementary file 4 [file Image4.PDF]

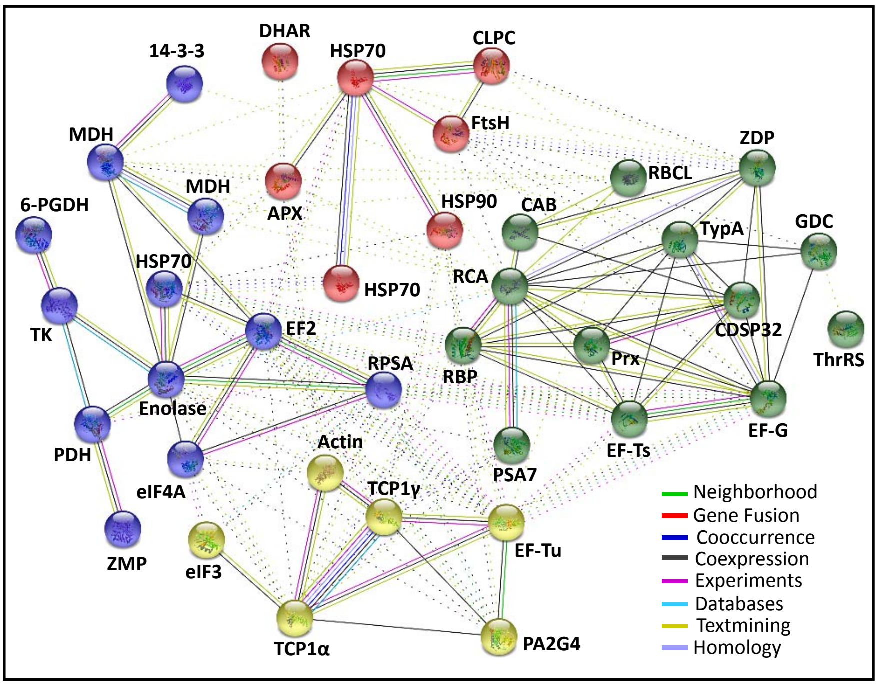

Supplementary Figure S4. The protein-protein interaction (PPI) network of proteins in *E. arvense* spore from STRING analysis in the evidence view. A total of 63 differentially abundant proteins represented by 38 unique homologous proteins from Arabidopsis are shown in PPI network. Nodes in different colors belong to four main groups. Different line colors represent the types of evidence for the association, including neighborhood, gene fusion, cooccurrence coexpression, experiments, databases, textmining, and homology. Detailed information on protein names and abbreviations can be found in Table 1.
