# Supplementary material for: Cytological and proteomic analyses of horsetail (Equisetum arvense L.) spore germination
Source: Front Plant Sci. 2015 Jun 17;6:441. doi: 10.3389/fpls.2015.00441 (PMC4469821; doi:10.3389/fpls.2015.00441)
Supplement: Supplementary file 7 [file Table3.DOC]

***Supplementary Table S3. List of proteins with multi-proteoforms in E. arvense spores.***

| **Protein name*^(a)^*** | **Spot No.*^(b)^*** | **Subcellular location*^(c)^*** | **Plant species*^(d)^*** | **Gi No.*^(e)^*** | **Thr. MW(Da)**  **/pI*^(f)^*** | **Exp. MW(Da)**  **/pI*^(g)^*** | **Cov (%)*^(h)^*** | **Sco*^(i)^*** | **QM*^(j)^*** | **V%±SD*^(k)^***  **MS RS DCS GS SPC** |
| --- | --- | --- | --- | --- | --- | --- | --- | --- | --- | --- |
| **Photosynthesis (15)** |  |  |  |  |  |  |  |  |  |  |
| Ribulose-1,5-bisphosphate carboxylase/oxygenase large subunit (RBCL) | 346 | Chl | *Eryngium bourgatii* | 1292976 | 53,093/  5.56 | 28,686/  6.28 | 6 | 59 | 3 | a  b  a  a  a |
|  | 763 | Chl | *Donatia fascicularis* | 1304292 | 49,896/  6.32 | 134,736/  5.48 | 8 | 55 | 5 | a  b  b  b  b |
|  | 902 | Chl | *Grammitis diminuta* | 340031166 | 45,938/  6.26 | 50,502/  6.31 | 5 | 61 | 2 | a  b  b  b  b |
|  | 117 | Chl | *Equisetum telmateia* | 16565336 | 48,750/  6.26 | 52,974/  6.47 | 15 | 57 | 7 | a  b  b  b  b |
|  | 196 | Chl | *E. telmateia* | 16565336 | 48,750/  6.26 | 51,502/  6.47 | 16 | 52 | 6 | a  b  b  b  b |
|  | 194 | Chl | *E. telmateia* | 16565336 | 48,750/  6.26 | 51,418/  6.33 | 13 | 53 | 6 | a  b  b  b  b |
|  | 400 | Chl | *E. telmateia* | 16565336 | 48,750/  6.26 | 49,452/  6.31 | 11 | 53 | 5 | a  b  b  b  b |
|  | 23 | Chl | *E. bourgatii* | 1292976 | 53,093/  5.56 | 50,872/  5.02 | 4 | 78 | 2 | a  b  b  c  d |
|  | 528 | Chl | *Equisetum arvense* | 1352773 | 52,493/  5.86 | 67,620/  4.64 | 7 | 60 | 3 | c  b  a  c  c |
|  | 100 | Chl | *Isoetes capensis* | 83032384 | 47,561/  6.30 | 42,425/  5.47 | 6 | 55 | 3 | a  a  b  a  b |
| Ribulose-1,5-bisphosphate carboxylase/oxygenase activase (RCA) | 34 | Chl | *Gossypium hirsutum* | 12620883 | 48,609/  5.06 | 48,999/  5.19 | 11 | 98 | 4 | a  d  cd  b  c |
|  | 184 | Chl | *Hordeum vulgare* | 100614 | 47,496/  5.64 | 47,356/  6.31 | 11 | 94 | 4 | ab  a  b  ab  ab |
|  | 125 | Chl | *Musa acuminata* subsp. *malaccensis* | 695062479 | 47,898/  6.18 | 42,859/  5.67 | 8 | 75 | 3 | a  b  e  d  c |
| Transketolase (TK) | 308 | Chl | *Spinacia oleracea* | 2529342 | 80,744/  6.20 | 95,817/  6.20 | 8 | 156 | 5 | b  c  a  c  a |
|  | 84 | Chl | *S. lycopersicum* | 460388792 | 80,615/  6.26 | 85,056/  6.11 | 8 | 90 | 7 | a  b  b  c  b |
| **Carbohydrate and energy metabolism (5)** |  |  |  |  |  |  |  |  |  |  |
| Malate dehydrogenase (MDH) | 160 | ^#^Chl, Cyt, Mit, Pox | *Arabidopsis thaliana* | 15219721 | 35,890/  6.11 | 46,411/  4.98 | 12 | 74 | 3 | b  ab  c  a  c |
| **Protein name*^(a)^*** | **Spot No.*^(b)^*** | **Subcellular location*^(c)^*** | **Plant species*^(d)^*** | **Gi No.*^(e)^*** | **Thr. MW(Da)**  **/pI*^(f)^*** | **Exp. MW(Da)**  **/pI*^(g)^*** | **Cov (%)*^(h)^*** | **Sco*^(i)^*** | **QM*^(j)^*** | **V%±SD*^(k)^***  **MS RS DCS GS SPC** |
|  | 503 | ^#^Chl, Cyt, Mit, Pox | *A. thaliana* | 15219721 | 35,890/  6.11 | 42,425/  5.53 | 6 | 57 | 2 | d  ab  bc  a  c |
|  | 260 | ^#^Chl, Cyt, Mit, Pox | *A. thaliana* | 11133509 | 35,548/  6.11 | 29,729/  6.41 | 6 | 59 | 3 | c  a  b  a  b |
|  | 164 | ^#^Chl, Cyt, Mit, Pox | *Beta vulgaris* subsp. *vulgaris* | 731361010 | 41,677/  5.74 | 36,353/  5.84 | 12 | 193 | 4 | b  a  c  c  b |
|  | 163 | Chl | *Brachypodium distachyon* | 357147942 | 41,864/  6.97 | 38,560/  5.94 | 10 | 60 | 4 | b  a  c  d  d |
| **Signaling and vesicle trafficking (4)** |  |  |  |  |  |  |  |  |  |  |
| Ran GTPase (RAN) | 276 | Nuc | *P. sitchensis* | 116794384 | 25,374/  6.30 | 27,727/  6.65 | 20 | 52 | 4 | c  b  a  b  b |
|  | 317 | Nuc | *Vicia faba* | 585783 | 25,274/  6.39 | 27,750/  6.73 | 27 | 187 | 6 | c  bc  ab  ab  a |
|  | 340 | Nuc | *P. sitchensis* | 116794384 | 25,374/  6.30 | 30,115/  5.59 | 24 | 53 | 5 | c  ab  a  ab  b |
|  | 764 | Nuc | *P. sitchensis* | 116794384 | 25,374/  6.30 | 44,900/  6.58 | 30 | 51 | 6 | c  bc  bc  b  a |
| **Cell structure (2)** |  |  |  |  |  |  |  |  |  |  |
| Reversibly glycosylated polypeptide (RGP) | 554 | Cyt | *Ricinus communis* | 223546230 | 41,557/  5.82 | 39,973/  5.41 | 14 | 105 | 5 | b  b  a  a  b |
|  | 118 | Cyt | *Solanum tuberosum* | 34582499 | 42,146/  5.71 | 35,827/  5.36 | 24 | 74 | 8 | a  b  ab  c  b |
| **Protein synthesis (4)** |  |  |  |  |  |  |  |  |  |  |
| Eukaryotic initiation factor 4A (eIF4A) | 66 | Cyt | *P. patens* | 168026095 | 47,119/  5.46 | 48,999/  5.32 | 25 | 162 | 11 | a  b  c  d  bc |
|  | 878 | Cyt | *Nicotiana. tabacum* | 1170511 | 47,098/  5.37 | 49,134/  5.23 | 14 | 118 | 6 | d  d  b  c  a |
| Elongation factor 2 (EF2) | 94 | Cyt | *O. sativa* subsp. *japonica* | 38344860 | 94,939/  5.85 | 115,425/  6.42 | 9 | 111 | 7 | b  b  c  c  a |
|  | 538 | Cyt | *S. moellendorffii* | 302773640 | 94,568/  6.00 | 114,195/  6.33 | 5 | 167 | 5 | a  c  b  c  a |
| **Protein folding and processing (5)** |  |  |  |  |  |  |  |  |  |  |
| Heat shock protein 70 (HSP70) | 542 | Cyt | *S. moellendorffii* | 302770212 | 71,931/  5.17 | 80,237/  5.18 | 13 | 295 | 7 | d  cd  a  b  bc |
|  | 1002 | Cyt | *Populus trichocarpa* | 224098390 | 71,620/  5.14 | 80,105/  5.21 | 16 | 135 | 8 | c  c  b  b  a |
| **Protein name*^(a)^*** | **Spot No.*^(b)^*** | **Subcellular location*^(c)^*** | **Plant species*^(d)^*** | **Gi No.*^(e)^*** | **Thr. MW(Da)**  **/pI*^(f)^*** | **Exp. MW(Da)**  **/pI*^(g)^*** | **Cov (%)*^(h)^*** | **Sco*^(i)^*** | **QM*^(j)^*** | **V%±SD*^(k)^***  **MS RS DCS GS SPC** |
|  | 1566 | Cyt | *Petunia* × *hybrida* | 20559 | 71,137/  5.07 | 106,146/  6.34 | 4 | 92 | 2 | a  b  b  b  b |
|  | 314 | Cyt | *Dactylis glomerata* | 188011548 | 72,002/  5.03 | 86,310/  5.38 | 21 | 338 | 13 | a  d  d  bc  c |
|  | 928 | Cyt | *S. lycopersicum* | 460394037 | 72,308/  5.16 | 91,122/  5.25 | 7 | 122 | 5 | a  b  b  b  b |
| **Protein degradation (5)** |  |  |  |  |  |  |  |  |  |  |
| Zinc dependent protease (ZDP) | a199 | Chl | *Trifolium pratense* | 84468286 | 74,746/  5.82 | 73,106/  5.66 | 12 | 106 | 7 | a  a  c  c  b |
|  | 227 | Chl | *T. pratense* | 84468286 | 74,746/  5.82 | 26,150/  5.94 | 10 | 87 | 7 | c  b  b  a  d |
|  | 37 | Chl | *T. pratense* | 84468286 | 74,746/  5.82 | 72,520/  5.57 | 5 | 93 | 3 | a  b  d  c  c |
| ATP-dependent zinc metalloprotease FtsH (FtsH) | 354 | Chl | *Micromonas pusilla* CCMP1545 | 303275720 | 77,421/  5.29 | 75,882/  5.26 | 6 | 113 | 4 | b  b  b  b  a |
|  | 508 | Chl | *P. patens* | 168001910 | 68,933/  5.23 | 108,174/  5.50 | 3 | 75 | 2 | c  c  a  b  c |
| **Stress and defense (5)** |  |  |  |  |  |  |  |  |  |  |
| 2-cys peroxiredoxin protein (Prx) | 414 | Chl, Cyt | *Erythranthe guttata* | 604334612 | 21,153/  4.98 | 21,812/  4.68 | 17 | 132 | 4 | a  a  a  b  a |
|  | 92 | Chl, Cyt | *Hyacinthus orientalis* | 47027073 | 21,956/  4.93 | 22,930/  5.24 | 12 | 58 | 3 | b  a  c  c  c |
| Dehydroascorbate reductase-like protein (DHAR) | 244 | Cyt | *S. tuberosum* | 76573291 | 23,610/  6.32 | 24,534/  5.60 | 11 | 147 | 3 | b  a  c  ab  a |
|  | 361 | Cyt | *S. tuberosum* | 76573291 | 23,610/  6.32 | 24,783/  5.64 | 11 | 105 | 3 | d  c  ab  bc  a  e  b  d  a  c |
|  | 899 | Cyt | *S. tuberosum* | 76160951 | 23,596/  6.09 | 44,783/  6.60 | 11 | 92 | 3 |  |

*^a^* The name and functional category of the protein proteoforms identified by ESI-Q-TOF and ESI-Q-Trap tandem mass spectrometry. The abbreviations for the protein names are indicated in the bracket after protein names. *^b^* Assigned spot number as indicated in Figure 3. *^c^* Protein subcellular localization predicted by softwares (YLoc, LocTree3, Plant-mPLoc, ngLOC, and TargetP). Only the consistent predictions from at least two tools were accepted as a confident result. Pounds (#) indicate prediction results were inconsistent among five tools. The subcellular localizations next to pounds were predicted based on literature listed in Supplementary Table S4. Chl, chloroplast; Cyt, cytoplasm; Gol, Golgi apparatus; Mit, mitochondria; Nuc, nucleus; Pox, peroxisome. *^d^* The plant species that the peptides matched from. *^e^* Database accession number from NCBI non-redundant protein database. *^f,g^* Theoretical (f) and experimental (g) molecular weight (Da) and pI of identified proteins. Theoretical values were retrieved from the protein database. Experimental values were calculated using ImageMaster 2D version 5.0. *^h^* The amino acid sequence coverage for the identified proteins. *^i^* The Mascot score obtained after searching against the NCBI non-redundant protein database. *^j^* The number of matched peptides for each protein. *^k^* The mean values of protein spot volumes relative to total volume of all the spots. Five spore germination stages, mature spores (MS), rehydrated spores (RS), double-celled spores (DCS), germinated spores (GS), and spores with protonemal cells (SPC) were performed. Error bar indicates ± standard deviation (SD). Letters indicate statistically significant differences (*p* <0.05) among five stages of spore germination as determined by one-way ANOVA.
